# Mechanisms of Film-Formation-Related Defects in EUV Photoresists for Sub-3 nm Nodes and Synergistic Materials–Process–Intelligence Co-Optimization

**DOI:** 10.3390/mi17070864

**Published:** 2026-07-21

**Authors:** Junlin Wu, Yanqing Luo, Junzhe Hu, Shirong Li, Sen Cai, Tiedong Cheng, Ping Zhang, Pei Li, Shengkun Jiang, Ziqiang Liu, Guitai Wu, Sergey Mikhailovich Kopytov, Jin Yang

**Affiliations:** 1 School of Electrical Engineering and Automation, Jiangxi University of Science and Technology, Ganzhou 341000, China; wujunlin2026@outlook.com (J.W.); chloeluo47@outlook.com (Y.L.); hujunzhe430@outlook.com (J.H.); limouren_1026@outlook.com (S.L.); c2548522868@outlook.com (S.C.); chengtiedong@126.com (T.C.); p.zhang@jxust.edu.cn (P.Z.); lipjob@163.com (P.L.); jsk7078@163.com (S.J.); 9120230071@jxust.edu.cn (Z.L.); 2 Department of Industrial Electronics, Komsomolsk-on-Amur State Technical University, Komsomolsk-on-Amur 681013, Russia; 3 Institute of Microelectronics of Chinese Academy of Sciences, Beijing 100029, China

**Keywords:** EUV lithography, sub-3 nm node, photoresist film-formation-related defects, stochastic effects, chemically amplified resist (CAR), non-chemically amplified resist (non-CAR), digital twin, virtual metrology, process control, closed-loop co-optimization

## Abstract

With the advancement of High-NA EUV lithography and the continued evolution of transistor architectures toward GAA and CFET, semiconductor manufacturing has entered the sub-3 nm technology node era. At advanced nodes, photon shot noise becomes increasingly significant, while the process tolerance window narrows substantially. Photoresist film-formation-related defects may originate from multiple stages of the fabrication process, including coating, exposure, post-exposure bake, development, and etching/stripping, and are strongly influenced by microscopic stochastic effects. However, the isolated optimization of materials, processes, or intelligent control strategies still suffers from significant limitations. Therefore, this review systematically examines the formation mechanisms and cross-process evolution of photoresist film-formation-related defects within the development trajectory of advanced lithography. An integrated materials–process–intelligence co-optimization framework is proposed to elucidate the coupling mechanisms among these three dimensions and the construction of a full-chain closed-loop control strategy. The current challenges and future development directions are summarized, providing optimization insights for both academic research and industrial implementation. This review aims to establish a defect-control framework integrating fundamental understanding with engineering considerations, thereby supporting low defectivity, high robustness, and improved manufacturability for sub-3 nm node patterning.

## 1. Introduction

With the iterative upgrades to High-NA EUV lithography systems and the gradual implementation of 3D transistor architectures such as GAA and CFET, semiconductor manufacturing has fully entered the sub-3 nm process era. As a key functional material in extreme ultraviolet (EUV) lithography, photoresists have become a major source of variability and defectivity at advanced nodes, and their defect behavior now strongly influences the pattern-transfer quality and final device yield [1,2,3]. To address these challenges, extensive efforts have been made from multiple fronts. At the materials level, researchers have sought to suppress stochastic defects and improve the line-edge roughness (LER) and critical-dimension (CD) variation by tailoring the molecular architecture, backbone rigidity, protecting-group design, and acid-diffusion behavior in photoresist systems [4]. At the process level, methods such as computational fluid dynamics (CFD) have been applied to optimize flow fields, wetting behavior, and film thickness uniformity during spin coating and related deposition steps, thereby mitigating film-formation-related defects and interfacial instabilities [5]. At the intelligence level, physics-constrained modeling, virtual metrology, and machine learning have been introduced to predict defect evolution and enable the closed-loop correction of process parameters [6,7].

Although substantial progress has been achieved in materials innovation, process optimization, and intelligent control for photoresist defect mitigation, the effectiveness of isolated optimization strategies is becoming increasingly limited in advanced manufacturing, where defect formation is governed by strongly coupled factors. A growing body of literature has therefore emphasized the importance of multidimensional collaborative optimization in semiconductor processing. For example, Kazazis et al. have noted in the context of EUV lithography that stochastic effects can only be effectively addressed through the co-optimization of photoresist materials and process conditions [8]. Similarly, Li and Aqad have emphasized that overcoming technological challenges at advanced technology nodes mandates the synergistic co-optimization between material systems and process conditions [9]. On the intelligent collaboration front, Kanarik et al. have demonstrated that human–machine collaboration can effectively enhance the efficiency of semiconductor process development, providing direct evidence for the feasibility of integrating process and intelligence in semiconductor manufacturing [10]. Related studies have further suggested that artificial intelligence will play an increasingly important role across the full lifecycle of materials design, synthesis, characterization, and application [11], a view also supported by Xia et al. [12]. Furthermore, the review by Zheng et al. has pointed toward the “material–process–intelligence” synergy as an important future direction for semiconductor manufacturing and research [13]. Taken together, these studies indicate that effective defect mitigation should not rely on isolated, single-factor improvements, but instead requires a systematic design based on the coupling among the material properties, process conditions, and intelligent methods.

Based on the above research progress, this review argues that the effective control of photoresist film-formation-related defects at advanced nodes can no longer be achieved through improvements in the materials, process conditions, or intelligent methods considered independently. Instead, it requires an integrated materials–process–intelligence framework together with a clear understanding of the framework’s engineering implementation pathways and practical boundaries. The structure of this paper is as follows: First, we trace the evolution of EUV lithography technology for sub-3 nm nodes from multiple perspectives—including optics, light sources, novel lithography schemes, and transistor architectures—to reveal the challenges posed by random photonic effects and the reduced process tolerance resulting from node scaling, and to demonstrate the necessity of controlling photoresist film defects. Next, it examines the defect generation mechanisms and inter-process evolution patterns for both CAR and non-CAR photoresists throughout the entire process flow, from coating to etching; we discuss the technical pathways, advantages, and limitations of the three dimensions of materials innovation, process optimization, and intelligent regulation, respectively, revealing the inadequacies of single-dimensional optimization. Then, we propose the core logic of an integrated materials–process–intelligence collaborative framework, discuss in detail the pairwise coupling pathways among materials, process engineering, and intelligent methods, and further describe the construction of a full-process closed-loop control system together with its engineering barriers and evaluation criteria. Finally, we summarize the key challenges, discuss future development trends, and provide targeted recommendations for academia and industry. The overall aim is to support the development of defect-control strategies with strong physical grounding, practical relevance, low defect density, high robustness, and excellent manufacturability for sub-3 nm patterning.

## 2. Frontiers in Extreme Ultraviolet Lithography and the Evolution of Sub-3-nm Nodes

### 2.1. Upgrades to the Optical System

Extreme ultraviolet (EUV) lithography with a numerical aperture (*NA*) of 0.33 and a wavelength of 13.5 nm is the mainstream patterning process in today’s advanced chip manufacturing. The minimum resolvable feature size in lithography is directly proportional to the exposure wavelength. EUV’s operating wavelength of 13.5 nm is far shorter than that of the 193 nm deep ultraviolet (DUV) light source. Leveraging the advantages of its short wavelength, EUV lithography has significantly reduced the resolvable half-pitch, thereby overcoming the patterning resolution bottleneck of traditional DUV lithography [14,15,16]. Currently, equipment of this specification has been deployed at major foundries such as TSMC, Samsung, and Intel for the mass production of 7 nm to 3 nm logic chips. The quantitative constraints on these two parameters are described by the Rayleigh diffraction limit equation:

(1) $$R=k_{1}\frac{\lambda }{NA}$$

A complete EUV lithography system consists of four core modules: the light source, the mask, the exposure stage, and a multilayer mirror assembly [17]. Currently, the mature, mass-produced models are the 0.33 *NA* specification; however, to further break resolution barriers and enable sub-3 nm and future process nodes, manufacturers are upgrading existing EUV platforms to High-NA EUV designs, driving lithography resolution to smaller dimensions. The numerical aperture represents the maximum light incidence angle striking the wafer surface; the quantitative relationship for this physical quantity is shown in Equation (2):

(2) NA=sinθ

The higher the value, the greater the resolution capability of the lithographic interference pattern. High-NA EUV increases the aperture of the end-of-wafer mirror, reconfigures the six-mirror folded optical path architecture, and adopts a half-field exposure scheme to increase the maximum beam tilt angle the system can accept, thereby raising the projection optical *NA* from 0.33 to 0.55 and further reducing the minimum lithographic resolution size *R*. This improvement represents an increase of approximately 66.7%, shrinking the minimum printable feature size by approximately 40%. It will bring multiple benefits to the semiconductor market, such as reducing the process complexity, improving yield rates, enhancing resolution, enabling finer pattern formation, increasing transistor integration density, and lowering technology costs. This will extend Moore’s Law for at least another decade [18,19,20].

Recently, ASML CEO Christophe Fouquet stated at an IMEC-hosted conference that the first mass-production devices based on next-generation High-NA EUV technology will be released in the coming months, covering both memory and logic chips. Intel is currently the most aggressive adopter of this technology: it is the world’s first customer to receive and integrate the Twinscan EXE:5000 High-NA EUV prototype, and had completed the deployment of multiple High-NA systems as of May 26 for advanced process development.

Hyper-NA EUV (Hyper-Numerical Aperture Extreme Ultraviolet Lithography) is the next-generation optical iteration in the 13.5 nm wavelength band, succeeding the 0.55 NA High-NA EUV. This technology optimizes the optical path based on the existing High-NA optical architecture, increasing the numerical aperture by widening the beam incidence angle, while also adjusting the multi-layer coating structure and introducing polarization control measures to mitigate the imaging degradation caused by high-angle beams. Hyper-NA EUV is currently in the early stages of R&D; prototypes are expected to be unveiled after 2030, with mass production not anticipated until around 2035. Its technical maturity is significantly lower than that of High-NA EUV systems already delivered to customers [20,21].

### 2.2. Upgrades to the Light Source System

To suppress the generation of random defects, both academia and industry continue to explore next-generation light source technologies. Although the operating wavelength of EUV lithography—currently used in advanced manufacturing processes—is much shorter than that of DUV lithography, the energy of a 13.5 nm EUV photon is as high as 92 eV, far exceeding the 6.4 eV of 193 nm DUV. Under the same exposure dose conditions, the number of EUV photons reaching the photoresist layer is only 1/14 that of DUV; this extremely low photon count is highly prone to inducing various types of random defects [3,8,22,23,24].

The current mass-production EUV lithography uses a tin-droplet laser-produced plasma (LPP) light source, which relies on a CO_2_-MOPA laser combined with a pre-pulse and hydrogen debris removal scheme to generate 13.5 nm extreme ultraviolet light [25,26]; however, due to the limitations in the laser conversion efficiency, there is a bottleneck in the light source power. When the power is insufficient, the total number of photons received by the wafer is reduced, and the shot noise becomes more pronounced, significantly increasing the probability of stochastic defects in lithography. The industry is continuously upgrading light source hardware architectures to increase the output power and address this issue [27].

Free-electron lasers (FELs) and gas cluster targets represent two next-generation EUV light source candidates intended to replace the current tin-droplet laser-produced plasma architecture. Both technologies aim to break the existing power bottleneck by boosting the photon flux, which suppresses the photon shot noise and mitigates lithographic random defects. FELs generate kilowatt-scale, high-coherence, wavelength-tunable EUV radiation via particle accelerators without introducing metallic contamination; notably, one FEL facility can serve multiple lithography tools [28,29,30,31]. In the race to develop EUV free-electron lasers for mass production in the semiconductor industry, the U.S.-based company xLight is currently the global leader. Recently, xLight secured $150 million in Series C funding and plans to launch a prototype compatible with existing ASML lithography systems in 2028.

Gas cluster targets replace molten tin with clusters of inert gases, eliminating the damage to optical components caused by tin debris at the source and achieving a higher conversion efficiency. They offer two development paths: a 13.5 nm retrofit route and a 6.7 nm ultra-short-wavelength route, intended for upgrading existing production lines and next-generation lithography systems, respectively. Recently, the Russian Academy of Sciences achieved significant progress in this field by forming lithium–xenon nanoclusters from a mixture of lithium vapor and xenon gas. The research team excited the clusters using a 1030 nm ytterbium femtosecond laser, causing them to emit 6.7 nm extreme ultraviolet light [32,33].

### 2.3. Redesign of the Lithography System

Section 2.1 and Section 2.2 above primarily discussed high-numerical-aperture optical iteration and optimization schemes for next-generation light sources. BEUV (soft X-ray extreme ultraviolet lithography), a long-term prospective lithography technology with enormous development potential [34], completely departs from the conventional 13.5 nm EUV technical framework. This system differs fundamentally from EUV in terms of the photon energy, optical adaptation requirements, and photoresist materials. Simply replacing the light source or upgrading individual optical components cannot fully realize the resolution gains offered by shorter wavelengths; therefore, this requires a comprehensive and systematic redesign of the entire lithography system.

In terms of the light source architecture, BEUV has moved away from the reliance on tin plasma light sources found in traditional commercial EUV systems and is instead exploring entirely new solutions, such as high-energy free-electron lasers (FELs). By increasing the electron beam acceleration energy and optimizing the undulator structure, this approach can stably emit short-wavelength radiation at 6.5–6.7 nm; combined with high-order differential vacuum beamlines and active polarization control, it not only completely eliminates the risk of debris contamination associated with traditional tin-based light sources but also significantly improves the beam quality and output stability [35].

At the same time, to fully exploit these high-energy short-wavelength photons, the optical system requires a comprehensive redesign. The original Mo/Si multilayer coating stack is entirely replaced by novel lanthanum-based (La/B) reflective films tailored for the BEUV wavelength band. On this basis, researchers reoptimize the surface curvature, beam incidence angles, and optical routing of the illumination micromirror array and projection lens, effectively mitigating the diffraction and aberration challenges in short-wavelength imaging [36].

In terms of the overall ecosystem, BEUV has also undergone a systematic overhaul. A paper published by Johns Hopkins University in *Nature* explicitly states that conventional organic EUV photoresists cannot be used directly with BEUV and require significant chemical structural modifications [37].

However, although BEUV has theoretical advantages in terms of the resolution, the entire lithography system has not yet undergone engineering validation, and the industrial supply chain is completely nonexistent, making commercial implementation difficult in the short term.

### 2.4. Key Dimensions of Various Devices at the 3 nm Node and Below

As process technology enters the ultra-advanced stage of 3 nm and below, relying solely on lithography equipment upgrades is no longer sufficient to meet miniaturization demands, and the underlying architecture of transistors is undergoing a fundamental transformation. Currently, FinFETs remain the dominant technology for mass-produced 3 nm nodes. TSMC, the industry leader, continues to optimize the FinFET architecture for its 3 nm (N3/N3E) process out of consideration for yield stability and cost control; although Samsung has aggressively introduced the GAA (MBCFET) architecture at the 3 nm node, the industry generally regards the extreme miniaturization of FinFETs as a defining characteristic of the 3 nm era [38,39,40,41].

However, FinFETs’ electrostatic control capabilities are gradually reaching their limits, and the short-channel effect is becoming increasingly pronounced. GAA (Gate-All-Around), on the other hand, rotates the channel by 90 degrees and replaces it with horizontally suspended nanosheets or nanowires, allowing the gate to completely surround the channel from all sides, thereby significantly enhancing the electrostatic control and reducing the leakage current [42,43,44]. Consequently, the 2 nm and subsequent processes will fully transition to GAA (Gate-All-Around) transistors; starting with the 2 nm (N2) node, leading foundries such as TSMC, Samsung, and Intel will fully adopt the GAA architecture.

Looking ahead to future nodes such as 0.5 nm and 0.3 nm, the CFET (Vertically Stacked Complementary Field-Effect Transistor) has emerged as the core direction of evolution. By stacking NMOS and PMOS transistors vertically, this architecture overcomes the size limitations of planar layouts and further increases the integration density [45,46]. Research by the Chinese Academy of Sciences indicates that CFETs can scale the height of standard logic cells down to 4-Track and reduce the area of SRAM cells by more than 40% [47]; according to industry forecasts, CFETs will officially replace GAA in 2034 (A7 node) and remain in use through the A3 (0.3 nm) node in 2040.

The four key feature size metrics used to evaluate the level of advanced process technology are as follows: the gate length (Lg), contact-to-gate spacing (CGP/CPP), minimum sub-pitch of the bottom metal layer, and transistor integration density. Combining the current commercially mass-produced measured data with R&D forecasts for next-generation advanced processes, the typical parameters for each process node are summarized in Table 1.

### 2.5. Challenges Posed by Continuous Process Node Scaling

#### 2.5.1. Yield Constraints Caused by Photolithographic Random Effects

Under EUV exposure conditions, the number of effective photons per unit area is limited, and the count of incident photons follows a Poisson distribution [49]. Therefore, for a given dose *D*, when the light of wavelength *λ* strikes a plane with area *A*, the average number of photons *N* is given by the following:

(3) $$N=\frac{DA\lambda }{hc}$$

where *h* is Planck’s constant; and *c* is the speed of light in a vacuum [3]. Since photons exhibit quantum discrete behavior, the actual number of incident photons fluctuates randomly around the average value, and the absolute standard deviation of the photon count is as follows:

(4) $$\sigma_{N}=\sqrt{N}$$

Corresponding to a relative dose fluctuation of

(5) $$\frac{\sigma_{N}}{N}=\frac{1}{\sqrt{N}}$$

As can be seen from the relative fluctuation formula, scaling down the feature size reduces the exposure area A and lowers the average number of photons *N*, while simultaneously increasing the relative photon noise. Due to the power limitations in mass-produced EUV light sources, it is difficult to suppress noise by increasing the dose. At sub-3 nm nodes, the gate and via areas are halved compared to 5 nm, significantly exacerbating the shot noise and inducing random defects such as CD random shifts, line edge roughness (LER), and via missing holes or bridging [50].

#### 2.5.2. Physical Process Tolerance Margins Are Significantly Reduced

For sub-3 nm nodes, the 0.55 High-NA EUV lithography system has a depth of focus far shallower than that of conventional 0.33 NA equipment, requiring the use of an ultra-thin photoresist to eliminate oblique-incidence shadows and ensure the pattern transfer quality [51]. Ultra-thin photoresist layers can capture fewer EUV photons, further exacerbating the roughness and line break defects caused by random photon fluctuations; at the same time, thin photoresists have poor etch resistance, and coating defects, pinholes, and impurity agglomeration can all directly cause pattern damage, significantly reducing the process tolerance [52].

On the other hand, mass production at the sub-3 nm node employs GAA nanostrip devices, while the 1 nm node will introduce CFET vertical stacking structures; three-dimensional devices, multilayer thin films, and back-side power supply processes significantly increase the wafer surface topographical variations. The depth-of-focus margin for High-NA EUV is extremely small, making even minute wafer steps and film thickness deviations highly prone to causing defocusing and pattern distortion [53].

Ultra-thin photoresists and complex 3D devices create dual constraints, drastically narrowing the process tolerance window across the entire flow. Even minor deviations in any process step can lead to device failures such as leakage, threshold drift, and interconnect breaks, necessitating an atomic-level control over the process uniformity, equipment, and lithography materials.

### 2.6. The Necessity of Controlling Photoresist Film Defects

GAA/CFET transistors for future nodes are rapidly evolving toward complex three-dimensional architectures, while the introduction of High-NA EUV technology is driving photoresist films toward extreme thinness. Against the backdrop of node scaling causing stochastic effects to intensify exponentially, the comprehensive restructuring of lithography systems faces enormous challenges. Therefore, while ensuring that ultra-thin photoresist films provide a good step coverage and can conformally coat the entire three-dimensional GAA/CFET topography, improving the overall flatness and thickness uniformity of the photoresist film can significantly suppress defects caused by photonic and chemical stochastic noise, thereby reducing the loss in chip yield due to stochastic effects. This approach represents the most feasible and cost-effective foundational optimization strategy for the mass production of High-NA EUV at sub-3 nm nodes. Consequently, controlling photoresist film defects is a practical necessity [54].

Furthermore, the “Advanced EUV Resist Patterning with Metal Oxide Resist (MOR)” technical presentation released at the Tokyo Electron EUVL Workshop in 2022 proposed that photoresist systems for the second-generation CFET node at the 0.7 nm technology node should adopt a hybrid resist scheme combining chemically amplified resists (CARs) with metal oxide resists (MORs). The resist thickness is expected to be controlled below 20 nm. Meanwhile, the exposure dose should be maintained below 40 mJ/cm^2^, with the target line-width roughness (LWR) after etching below 1.8 nm and single-bridge defect density below 0.1 cm^−2^. The metal contamination level of the resist should be controlled below 1 × 10^10^ atoms/cm^2^. The system is also expected to support negative-tone development and be compatible with the ESPERT™ development process to reduce the exposure dose, and prevent pattern collapse and missing contact holes, while maintaining excellent etch resistance for multilayer pattern transfer in CFET structures.

Future-generation GAA and CFET transistors are rapidly evolving toward increasingly complex three-dimensional architectures, while the introduction of High-NA EUV lithography further drives photoresist films toward ultrathin configurations [55]. Under the condition that stochastic effects are significantly intensified during continued technology scaling [3], the comprehensive reconstruction of lithographic systems faces substantial challenges. Therefore, while ensuring that ultrathin photoresist films maintain sufficient step coverage and achieve conformal coating over three-dimensional GAA/CFET structures, improving the overall film uniformity and thickness control, as well as regulating related photoresist defects, can effectively suppress the defect formation induced by photon and chemical stochastic noise. This approach can reduce the impact of stochastic effects on the chip yield degradation and represents one of the most feasible and cost-effective fundamental optimization strategies currently considered for High-NA EUV manufacturing at sub-3 nm technology nodes. Therefore, controlling photoresist film-formation-related defects is of significant practical importance [56].

## 3. Mechanisms of Photoresist Film Defects

Building on an understanding of the significance of research into photoresist film defects, further elucidating the underlying mechanisms of photoresist film defects is a core prerequisite for achieving precise control over these defects.

Currently, mainstream EUV photoresists are primarily divided into two categories: chemically amplified photoresists (CARs) and non-chemically amplified photoresists, as represented by ZEP520A. Differences in the composition of these systems directly result in variations in their defect evolution behavior and process tolerance [57].

Chemically amplified photoresists are multi-component systems comprising a base resin, a photoacid generator, a quencher, and levelling agents; they can mitigate certain topographical fluctuations from front-end processes. After exposure and during PEB, photoacids diffuse within localized regions and trigger catalytic chemical reactions. Combined with the thermally driven resin segment motion, this process constitutes the primary source of stochastic defects [58]. In contrast, non-chemically amplified photoresists do not adopt a photoacid-catalyzed amplification mechanism; their imaging relies directly on exposure-induced chemical transformations such as main-chain scission or crosslinking. No acid-driven amplification occurs, so defect formation is mainly governed by the initial film quality and exposure fluctuations [59].

Although defects in both chemically amplified photoresists and non-chemically amplified photoresists can occur throughout all stages—including cleaning, coating, exposure, development, and subsequent pattern transfer—the dominant sensitivity mechanisms differ between the two. CAR is highly sensitive to the post-exposure heat treatment process, particularly the acid diffusion and chemical reaction completeness triggered by PEB; therefore, its defects often exhibit significant evolution between the post-exposure and development stages [60]. In contrast, non-CAR does not rely on acid-catalyzed amplification, and its defects are primarily influenced by the initial film quality, the direct effects of exposure, and the stability of development and pattern transfer. Based on these differences, this chapter will systematically analyze the mechanisms underlying film formation defects in these two types of photoresists, taking into account the complete photoresist process flow.

### 3.1. Photoresist-Related Supporting Processes

Substrate pretreatment, photoresist spin coating, soft baking, exposure, post-exposure baking (PEB), development, plasma etching, and residue removal constitute the complete basic process flow for the photolithography process using chemically amplified photoresists. For non-chemically amplified photoresists, PEB is not a mandatory step, and the pattern transfer path after development differs to some extent. The following sections will analyze the mechanisms behind film formation defects in both types of photoresists layer by layer, following the aforementioned process sequence; this section, however, provides a systematic overview of the current state of the art in the photoresist process field, both in academia and industry.

In addition to the coating process, pre-baking (PAB) and post-exposure baking (PEB) are also critical steps that determine the structure of the photoresist film and the quality of the pattern. Pre-baking primarily influences the subsequent exposure process by removing residual solvents, enhancing film stability, and improving substrate adhesion, while post-exposure baking (PEB) further determines the spatial distribution of photochemical reactions after exposure. For chemically expanded photoresists, the photoacid generated during exposure must diffuse during the PEB process to catalyze the removal of protective groups; however, excessive acid diffusion can lead to blurred pattern boundaries and increased line edge roughness (LER). Consequently, the current research is increasingly focusing on temperature uniformity, heating and cooling rates, and environmental condition control during the baking process. By regulating the solvent migration, molecular chain segment motion, and photochemical acid diffusion behavior, researchers aim to achieve a balance between resolution, sensitivity, and defect control.

Regarding the development process, as feature sizes continue to shrink, the impact of the development process on the final structure quality is becoming increasingly significant. In the traditional TMAH development, the concentration of the developer, development time, and mass transfer behavior at the interface can all cause local dimensional deviations and pattern defects. Consequently, advanced development processes are evolving toward higher selectivity and lower defect rates by optimizing the development conditions and controlling the dissolution kinetics during development to enhance the stability of the pattern transfer process.

At every stage of the photoresist process, there is a potential for defects to be induced in the photoresist film. The following section provides a detailed analysis of the defect formation mechanisms for chemically amplified photoresists and non-chemically amplified photoresists at each process step.

### 3.2. Substrate Preparation, Photoresist Coating, and Soft-Bake Stages

Many film-formation-related defects do not originate during exposure, but are already seeded at the early stage when the photoresist first contacts the substrate and forms a continuous liquid film. Chemical stochasticity arising from structural non-uniformities in the resist material may emerge as early as the coating stage, thereby establishing the initial conditions for subsequent defect propagation and amplification [3]. Relevant studies on spin-coating processes and fluid dynamics have shown that surface topography, roughness, trenches, and step structures all affect the final film thickness distribution [61,62,63,64], and can induce liquid retention, insufficient backflow, and edge enrichment [65].

**Figure 1:**
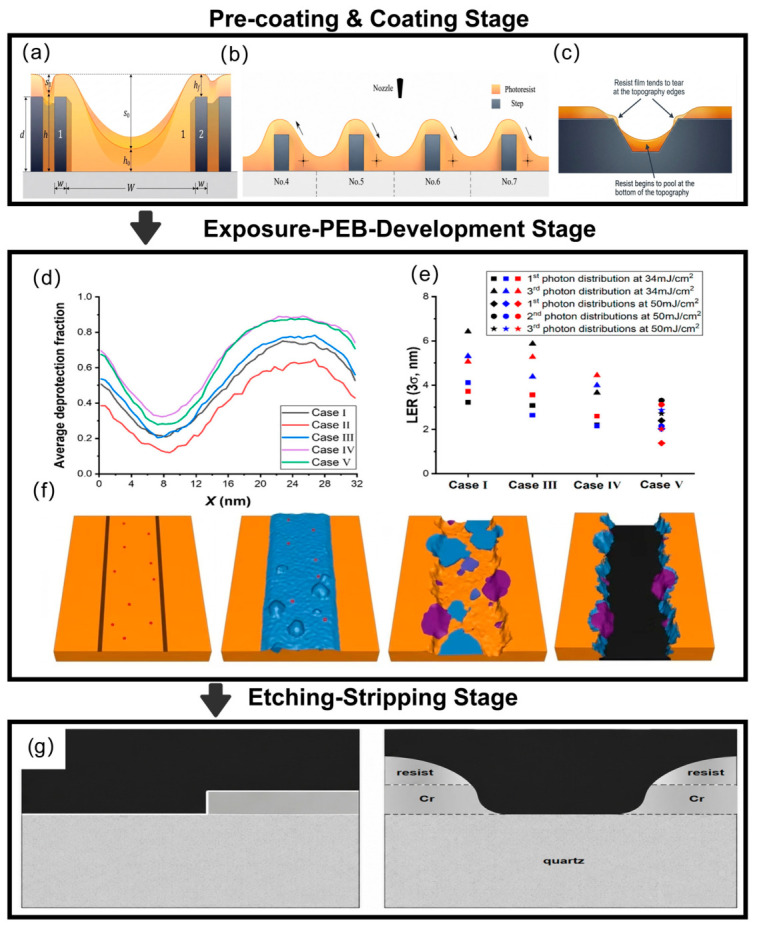
Typical defect morphologies and evolution characteristics throughout the photolithography process. (**a**) Static morphology of the photoresist at high-step structures on a wafer; the step structures cause inherent thickness variations in the photoresist, leading to initial film thickness non-uniformity defects. Adapted from [62]. (**b**) Dynamic flow morphology of photoresist as it flows over high-step structures during spin coating; the differential movement of the fluid creates a “half-side effect”, exacerbating film thickness non-uniformity across the entire wafer. Adapted from [62]. (**c**) Non-uniform photoresist coating morphology in step regions; defects such as edge discontinuities, photoresist accumulation in grooves, and undercutting occur due to the combined effects of gravity and surface tension. Adapted from [69]. (**d**) Curve showing the proportion of lateral deprotection of photoresist after PEB baking, comparing five sets of operating conditions (Case I–V) [73] ©2023, MDPI (CC BY 4.0). (**e**) Quantitative statistics on line edge roughness (LER) of formed lines under each set of operating conditions [73] ©2023, MDPI (CC BY 4.0). (**f**) Evolution of the photoresist surface morphology during development. Adapted from [74]. (**g**) Comparison of ideal etching versus actual etching. Adapted from [75].

In particular, researchers have conducted a morphological characterization of large-topography step structures on wafers and have established a theoretical model of photoresist film formation over high-step structures, with the specific structural morphology and film distribution features illustrated in Figure 1a [62]. This model defines several key structural and film thickness parameters, including step height *d*, step width *W*, trench width ω, as well as photoresist thicknesses in different regions: *h*_0_ (exposed pattern area), *h*_1_ (step gap), *h_f_* (standard thickness on flat substrate), *s*_0_ (maximum step height), and *s*_1_ (minimum step height). These parameters satisfy the following geometric relationship:

(6) $$h_{0}+s_{0}=h_{1}+s_{1}=h_{f}+d$$

Based on this model, researchers have introduced the degree of planarization (DOP) as a metric for quantitatively characterizing the surface planarity of the photoresist film after coating, which is calculated as follows:

(7) $$DOP=100\times \frac{h_{0}-h_{f}}{d}\;(\%)=(1-\frac{s_{0}}{d})\times 100\;(\%)$$

This metric enables the quantitative evaluation of the ability of a photoresist to fill high-topography substrate features and provides an intuitive measure of the planarization performance after coating. Both experimental measurements and simulations indicate that complete planarization over high-step structures is generally not achieved. Instead, only partial filling occurs, leaving residual microscopic height variations on the film surface. As a result, microscopic defects in the form of thickness non-uniformity are inherently embedded in the film at the structural level [62].

From a hydrodynamic perspective, the spin-coating process is governed jointly by centrifugal force, viscous resistance, solvent evaporation, and surface tension [66]. If the local solvent evaporation rate exceeds the film leveling rate, the photoresist liquid film may solidify prematurely before the thickness distribution has fully homogenized, thereby permanently retaining the initial thickness non-uniformity [67]. Meanwhile, the mainstream spin-coating process employs center-dispensing and radial spreading, where the liquid photoresist flows from the wafer center (exposure unit No. 5) toward the edge. During this flow, high-step and low-step structures impose different degrees of obstruction on the moving liquid. Specifically, the front-side steps (No. 4 and No. 5) exhibit significantly stronger flow obstruction than the rear-side steps (No. 6 and No. 7), resulting in resist accumulation and thickening in the front-side regions and thinning in the rear-side regions—a phenomenon known as the “half-side effect.” Such dynamically induced flow non-uniformity further amplifies the influence of the initial substrate topography and creates the conditions for a subsequent critical-dimension (CD) shift [68]. The flow variation characteristics of the photoresist are illustrated in Figure 1b. At the sub-3 nm advanced technology node, even such thickness deviations that are macroscopically imperceptible can induce a significant CD shift, compromising the lithographic precision [62].

Under the combined action of centrifugal force, gravity, and surface tension, the resist near step edges tends to undergo stretching-induced thinning or even local rupture, whereas trench regions are prone to excessive resist accumulation. These effects can ultimately produce coating-related defects such as step-edge cracking and trench-bottom erosion. The resulting non-uniform film morphology at step locations is shown in Figure 1c [69].

In addition, common coating defects, including edge beads, microbubbles, and pinholes, should not be regarded as independent random events. Rather, they are systemic manifestations of the film-formation instability caused by the coupled effects of the resist viscosity distribution, solvent composition, and ambient temperature and humidity [70]. Overall, the existing studies on coating processes indicate that photoresist film formation in advanced manufacturing must satisfy not only the basic requirement of film continuity, but also strict requirements for global thickness uniformity, stable film properties, controllable microscopic surface defects, and a reliable subsequent photochemical response. These requirements become particularly critical to yield and process stability at the sub-3 nm technology node [71].

At this stage, because the CAR photoresist system contains leveling agents and plasticizing additives, the resin molecules exhibit greater fluidity during the spin-coating process, allowing some defects to self-heal; as a result, the probability of defects being carried over to downstream processes is relatively low; in contrast, non-CAR single-component additive-free systems—such as ZEP520A—lack a molecular relaxation capability. Defects induced during the front-end process are difficult to repair in situ, and pre-baking further solidifies the film, causing these defects to often carry over into subsequent process steps [72].

Therefore, photoresist film formation in advanced processes—particularly in non-CAR systems—must not only meet the basic requirement of film continuity but also ensure uniform film thickness across the entire area, stable film performance, and controllable microscopic surface defects, while simultaneously guaranteeing stable and controllable subsequent photochemical reactions. This process requirement has a particularly critical impact on the lithography yield and process stability at sub-3 nm advanced process nodes [71].

### 3.3. Exposure–Post-Exposure Bake–Development Stage

Exposure, PEB, and development are the critical stages where the morphology and dimensional defects in chemically amplified photoresists are primarily generated. These defects originate from the coupled effects of photoacid generation, acid diffusion, and catalytic deprotection, which are characteristic of CAR systems. Such formation defects, which arise from acid diffusion imbalances, have a relatively minor impact in non-chemically amplified photoresist systems—such as ZEP520A—that lack PAG and acid-catalyzed mechanisms [4].

At this stage, the fundamental cause of defects is that the extent of the chemical reaction varies significantly across different locations following exposure and post-exposure baking (PEB). Regions that undergo a sufficient reaction are more readily dissolved during development, whereas insufficiently reacted regions remain less soluble. This spatial non-uniformity in dissolution behavior increases the pattern-edge roughness, raises the defect counts, and ultimately degrades the device yield [1,76,77].

In EUV lithography, photons, photoelectrons, and secondary electrons exhibit intrinsic stochastic characteristics, resulting in randomness in energy deposition and photoresist chemistry, which leads to an intrinsic stochasticity in both energy deposition and photoresist chemistry. Relevant experiments have employed multiple exposure dose gradients, with observation windows of 64 nm horizontally and 32 nm vertically. Using a blue-to-red color scale corresponding to indicator fractions from 0% to 100%, the reaction extent, dissolution behavior, and depth-dependent resist fraction were statistically analyzed under different exposure doses. Under all tested conditions, the measured indicators exhibited scattered distributions, directly reflecting the non-uniformity of the photochemical response. Representative results are shown in Figure 1d [78].

For chemically amplified resists (CARs), latent-image formation is governed by the coupled sequence of photon absorption, acid generation, acid diffusion, and deprotection reactions [79]. If the coated film exhibits thickness non-uniformity or spatial variations in the distribution of photoacid generators (PAGs), quenchers, and other components, the reaction efficiency can vary locally even under identical exposure conditions. Residual solvent content can further aggravate such non-uniformities. Such microscopic reaction non-uniformities readily lead to severe issues including residue, pattern bridging, and line breaks [80].

PEB is primarily used to regulate acid diffusion and drive the completion of the chemical reaction. A study combined the distribution curves of laterally deprotected groups after PEB under different process conditions (Figure 1d) with the statistical analysis of line-edge roughness (LER) shown in Figure 1e, demonstrating that PEB-induced heating drives the lateral diffusion of photoacid. This not only amplifies the inherent microscopic inhomogeneities caused by the random distribution of photons during the exposure stage but also exacerbates the discrete distribution of deprotected molecules at the exposure boundary, thereby smoothing out the concentration difference of deprotected groups between the exposed and unexposed regions, ultimately leading to a direct deterioration in the edge roughness of the photolithographically formed pattern [81].

In chemical amplification photoresist systems, development is also a critical step in which minute defects become significant problems. Since the dissolution difficulty varies by location—coupled with photoresist swelling, particle detachment, and surface residue—what were originally subtle unevenness issues can evolve into defects such as pattern adhesion, line breaks, residual photoresist at corners, and hole deformation [73]. In related experiments, the surface topography of 32 nm isolated lines in an acrylic-based SSR4 photoresist following EUV exposure was observed throughout the development process using high-resolution atomic force microscopy. It can be observed that thickness variations in the photoresist film were already present before development. As development progressed, microscopic defects continued to magnify, gradually leading to issues such as surface roughness, localized swelling, and film damage, ultimately resulting in macroscopic defects such as line defects. The evolution of the surface topography is shown in Figure 1f; this experiment clearly demonstrates the non-uniform swelling characteristics of the photoresist film; the uneven top corners and edges of the lines are also the direct causes of line width roughness, pattern distortion, and dimensional shifts [73].

For high-aspect-ratio, narrow-line-width structures, capillary forces generated during drying after development can also cause pattern collapse; therefore, the development stage is also a high-risk phase for structural mechanical instability [74]. Consequently, development is not merely a simple process of washing away excess photoresists; rather, it is a process that concentrates and magnifies all issues arising from the spin-coating, exposure, and reaction stages.

Non-chemically amplified photoresists, such as ZEP520A, do not contain photoacid generators or acid-catalyzed reaction systems; instead, they rely primarily on high-energy radiation to directly cleave the polymer backbone, thereby achieving differential dissolution during development. There are no amplified reactions involving the generation, diffusion, and deprotection of photogenerated acids; thus, defects such as edge roughness degradation and blurred exposure boundary gradients induced by acid diffusion can be effectively avoided [4]. The discrete nature of EUV photons still causes microscopic randomness in polymer chain breakage; combined with film thickness and molecular weight distribution variations, this can result in slight dissolution differences. However, since there is no chain-reaction amplification effect, the overall degree of non-uniformity is significantly lower; post-exposure baking, when applied, mainly serves to remove residual solvents and relieve internal film stress rather than inducing acid-catalyzed amplification reactions; it does not amplify microscopic defects. Linewidth shifts may occur only when baking is excessive, potentially causing the thermal degradation of polymers in unexposed areas. At the same time, the development process for this type of photoresist involves minimal swelling, resulting in fewer pattern distortions and edge defects. Residual photoresist is caused solely by insufficient polymer cleavage due to under-exposure; the only issue consistent with chemically amplified photoresists is capillary collapse during drying in structures with high aspect ratios. Overall, non-chemically amplified photoresists have fewer defect-inducing factors, exhibit no significant defect amplification behavior, and offer superior pattern formation stability.

### 3.4. Etching–Stripping Stage

However, during the etching and stripping stages of both photoresist systems, the defects generated in previous processes do not disappear; instead, they continue to evolve and become transferred into the final pattern. In experiments examining the cross-sectional morphology of pristine chromium films underlying chromium films before etching, both sample groups exhibited sidewall angles close to 90°, with vertical and well-defined profiles free of obvious distortion or over-etching. These profiles therefore served as a reference for evaluating the etching performance [75].

Once etching begins, regions protected by thinner resist regions lose their masking capability first, whereas thicker regions or residual resist may create excessive shadowing. This non-uniform protection results in dimensional errors and even a structural fracture. At the same time, the resist sidewalls are subject to erosion and deformation. These morphological changes and lateral losses are then transferred directly to the underlying chromium film, producing over-etching phenomena such as lateral etching and bottom undercut. Consequently, the sidewall profile, linewidth, and trench morphology deviate from their intended shapes. A comparison between the ideal and actual etching is shown in Figure 1g [75,82].

If the photoresist inherently exhibits weak adhesion or contains residual internal stress, ion bombardment during the plasma etching process can further damage the film surface layer and interfacial microstructure, thereby exacerbating interfacial damage [83].

The subsequent step is photoresist removal. Previous high-energy processes, such as ion implantation, can induce photoresist cross-linking and hardening, resulting in a stronger bond between the photoresist film and the underlying material, which significantly increases the difficulty of removal. If the photoresist is not completely removed, the residual organic material will continue to interfere with subsequent processes, affecting the overall process stability [84]. As can be seen, etching and stripping are not merely simple post-processing steps; they are critical stages that ultimately determine the final form of all defects created in earlier stages. They not only affect whether the pattern can be faithfully reproduced but also impact residual contamination, interface damage, and the overall reliability of the device.

### 3.5. Summary of This Chapter

This chapter provides a comprehensive analysis of the evolution of photoresist film-formation-related defects throughout the entire fabrication process for both CAR and non-CAR photoresists, as represented by ZEP520A. These defects occur throughout the entire process, from spin coating to exposure–PEB–development to etching and stripping.

During the spin coating stage, initial defects such as uneven film thickness arise from substrate steps and spin-coating fluid effects; CAR photoresists, which contain leveling additives, possess a certain degree of self-healing capability, whereas defects in single-component non-CAR photoresists are prone to carry over to subsequent processes. The exposure–PEB–development stage is where the differences between the two types of photoresists are most pronounced: CAR photoresists are subject to the random effects of EUV photons coupled with acid diffusion, and the PEB process amplifies microscopic inhomogeneities, leading to defects such as LER degradation, residual photoresist, and pattern distortion; non-CAR resins rely on radiation-induced chain scission for imaging and lack an acid-catalyzed amplification effect; they exhibit fewer defects and better formation stability, with pattern collapse due to capillary forces being the only common mechanical defect. The etching and stripping stage solidifies all defects from earlier stages; uneven film thickness, sidewall erosion, and stripping residues will cause over-etching of the underlying film, dimensional shifts, and interface damage. At sub-3 nm EUV nodes, random photon effects intensify, the process tolerance window narrows, and the difficulty of suppressing and precisely controlling various defects increases significantly [23,85,86].The two mechanisms of photoresist defects elucidated in this chapter provide a fundamental basis for subsequent defect mitigation strategies.

## 4. Materials Innovation and Breakthroughs for Addressing Photoresist Film-Formation-Related Defects

Photoresist patterning defects have become a key area of research in the field of micro- and nano-lithography materials; the design of photoresist materials themselves is the fundamental approach to suppressing various types of patterning defects. At present, the two most representative technological routes are the optimization and upgrading of chemically amplified resists (CARs) and the development of non-chemically amplified resists (non-CARs).

In Table 2 below, the performance results of the most promising photoresist materials are summarized. These materials are arranged according to their design principles and the main component types in the photoresist formulation. The performance metrics include the best reported resolution and the corresponding exposure dose; the pattern quality data (i.e., LER/LWR: line edge roughness/line width roughness) are also provided where available [87].

### 4.1. Mechanisms and Constraints of Chemically Amplified Resist Materials

At advanced technology nodes, the focus of materials innovation is no longer merely on improving the resolution and sensitivity. More critically, under fixed exposure dose and process window constraints, the goal is to suppress stochastic defects as much as possible and to render subsequent development and pattern transfer processes less sensitive and more robust against local microscopic variations [17,122]. Therefore, the evaluation criterion for materials design should shift from single-performance optimization to a comprehensive balance between defect risk and manufacturability.

Chemically amplified resists (CARs) remain the most mature and high-volume-manufacturing-ready mainstream system in advanced lithography to date, and their long-standing dominance is built upon the combined advantages of high sensitivity, high throughput, and mature process compatibility [9]. The fundamental principle of CAR is that a small number of exposure events can trigger the generation of acid from a photoacid generator (PAG); the acid then catalyzes the deprotection of protecting groups or crosslinking/degradation reactions during the post-exposure bake, thereby achieving signal amplification with one exposure and multiple chemical transformations [123]. This mechanism allows CAR to drastically lower the exposure doses, a key advantage already realized in the DUV era and to be smoothly integrated into standard process flows such as spin-coating, baking, development, and etching, making it one of the most industrially mature photoresist systems.

However, in the context of advanced EUV lithography, the CAR’s unique system of photochemical acid generation, diffusion, and catalytic amplification reveals inherent limitations that are difficult to avoid. Random intrinsic noise caused by the discreteness of EUV photons can induce patterning defects, while the acid-thermal diffusion driven by PEB heat treatment further amplifies these microscale inhomogeneities [122]. The high PAG loading and strong catalytic amplification design adopted to enhance lithographic sensitivity simultaneously amplify random acid distribution deviations, exacerbating the edge roughness and random defects. Consequently, CAR optimization remains unable to break through the RLS trade-off bottleneck—the interplay between sensitivity, resolution, and line edge roughness.

Constrained by its intrinsic imaging mechanism, the optimization of individual components in CAR invariably entails significant performance trade-offs. Enhancing the PAG performance to improve the acid generation efficiency aggravates the acid diffusion and degrades the pattern profile [79,124]. Strengthening the acid diffusion suppression can improve LER metrics, but at the cost of a reduced lithographic sensitivity and a narrowed process window [125]. Likewise, improper quencher loading can cause insufficient acid utilization and incomplete development [79]. These observations demonstrate that formulation optimization alone cannot resolve the core contradiction in which stochasticity and signal amplification are inherently coupled in CAR systems.

Therefore, a more reasonable assessment of CAR is warranted. In the foreseeable future, they will remain the dominant industrial system, possessing irreplaceable practical value, particularly in mature nodes, certain EUV nodes, and scenarios requiring a high compatibility with existing process flows. However, in the long run, their performance limits are bounded by their intrinsic imaging mechanisms [126].

### 4.2. Advantages and Challenges of Non-Chemically Amplified Resist Materials

To mitigate the classic trade-off among resolution, line-edge roughness, and sensitivity in CARs, increasing attention has been directed toward non-chemically amplified resist systems (non-CARs) that do not rely on long-range chemical amplification [127]. These systems leverage shorter reaction chains to reduce the negative impact of acid diffusion, and exploit the molecular structure design to enhance the reaction localization, thereby improving the pattern fidelity. Unlike CARs, which depend on acid generation, diffusion, and catalytic amplification, non-CARs incorporate photosensitive units directly into the molecular structure, allowing photon irradiation to induce the solubility change more directly and thereby establishing a more immediate causal link between exposure and development [4,127]. Therefore, they are theoretically more favorable for reducing LER/LWR and stochastic distortions.

Various sub-categories—including molecular glass (MG), multi-trigger resist (MTR), hydrogen silsesquioxane (HSQ), and metal oxide resist (MOR)—each possess distinct technical characteristics. Among them, MG systems typically feature more well-defined molecular structures and a lower compositional dispersity, which are believed to improve the film formation uniformity and reduce the dimensional fluctuations caused by material non-uniformities [128]. By introducing a higher reaction threshold and a multi-event triggering mechanism, MTR systems are expected to improve the tolerance to EUV shot noise [129]. HSQ, owing to its crosslinking characteristics based on a silicon–oxygen network, typically exhibits high resolution potential and offers advantages in pattern stability [130]. MOR systems, in patterning applications, are highly dependent on a synergistic design with underlying layers and interfacial materials [131,132].

It should be noted, however, that non-CARs are not inherently superior to CARs, but rather provide alternative pathways to address different bottlenecks. Their advantages are mainly reflected in the more precise and localized reaction control, the effective reduction in edge roughness and scalloping, and cleaner pattern boundaries. However, these advantages may come at the cost of challenges such as a decreased sensitivity, stringent particle contamination control in the equipment, the complex removal of inorganic residues, and the increased difficulty in manufacturing integration [133].

Therefore, the value of non-CARs lies not in completely replacing CARs, but rather in offering differentiated solutions to specific bottlenecks. For advanced node manufacturing, what truly merits attention is not an abstract comparison of which approach is “more advanced,” but rather a clear definition of the applicable boundaries of different systems and their associated engineering costs.

### 4.3. Design Principles of Photoresist Materials for Sub-3 Nm Nodes

As semiconductor manufacturing advances toward sub-3 nm nodes, the design philosophy of photoresists has undergone a significant transformation. Traditional research has primarily focused on fundamental exposure performance metrics such as resolution, sensitivity, and exposure contrast. At advanced technology nodes, however, film formation stability, layer uniformity, and compatibility with the substrate and upstream/downstream processes have become critical factors influencing pattern-transfer fidelity. Accordingly, photoresist design strategies must shift toward the comprehensive regulation of the full-process stability.

From the perspective of the material structure, suppressing microscopic non-uniformities within the film is central to improving the overall performance of photoresists. A photoresist system typically consists of a polymer matrix, photosensitive components, and various functional additives, and the compatibility and diffusion behavior among these components directly affect the film formation quality [72]. An unreasonable molecular design can readily lead to component aggregation and phase separation, which, in ultrathin films, further degrade the exposure reaction uniformity and development consistency, thereby compromising the pattern fidelity. Therefore, optimizing the polymer backbone and side chain structures, matching the component polarity, and rationally selecting the additives are key approaches to mitigating film defects at the source.

The film-forming behavior of the photoresist during spin-coating and pre-baking also affects the final film quality. This process involves changes such as solvent evaporation, molecular chain rearrangement, and component migration [72]. An imbalance in the solvent system can lead to the uneven distribution of the residual solvent in localized areas, which further induces topographical distortions at sub-3 nm nodes. To address this, optimizing the solvent ratio and controlling the evaporation rate and pre-bake parameters can effectively improve the film’s spreading ability and curing uniformity [134,135].

In addition, the interfacial compatibility between the photoresist and the substrate cannot be overlooked. Substrate surface characteristics directly affect the wetting behavior and adhesion performance of the photoresist [136]. Poor interfacial bonding tends to induce film delamination, edge cracking, and stress-induced instability. Through interfacial regulation strategies—such as underlayer modification and surface treatment—a balance between adhesion and uniform film formation can be achieved, thereby reducing the probability of interfacial defects [137].

In summary, the photoresist design at the sub-3 nm node must not pursue exposure metrics in isolation, but rather comprehensively integrate full-process factors including the film formation kinetics and interfacial properties. Future photoresist research and development will also need to be deeply integrated with process engineering and intelligent control approaches to synergistically achieve defect control objectives [138].

### 4.4. Summary of This Chapter

In summary, materials innovation plays a critical role in photoresist defect control. At the sub-3 nm node, whether for traditional chemically amplified resist (CAR) systems or emerging non-chemically amplified resist (non-CAR) systems, the evaluation criteria should move beyond the limitations of single-exposure performance optimization toward the comprehensive regulation of the full-process stability. This requires that future photoresist materials design consider not only the performance of the photoresist itself, but also the influence of relevant environmental factors, thereby reducing film-formation-related defects at the source.

However, it must also be clearly recognized that the role of materials innovation has boundaries. While it can effectively reduce the probability of defect occurrence, it cannot single-handedly eliminate subsequent failures caused by process disturbances, environmental drift, and cross-step amplification effects. Particularly under the stringent requirements of the sub-3 nm node, relying solely on the material dimension is often insufficient to completely resolve the challenges of photoresist defect control. Therefore, the continued development of future photoresist materials cannot be confined to the optimization of the material itself, but must also integrate other relevant factors such as process and intelligence. Through multi-dimensional synergistic efforts, the effective control of photoresist defects can be achieved, providing reliable support for advanced node patterning manufacturing.

## 5. Process Optimization and Upgrading for Addressing Photoresist Film-Formation-Related Defects

If materials design defines the intrinsic boundaries of defect control, then the task of process optimization is to determine whether the potential of these materials can be reliably realized under actual manufacturing conditions.

### 5.1. Upgrading of Coating Processes

Currently, spin coating remains the mainstream technology for photoresist film formation. However, for sub-3 nm nodes, where ultrathin resist films and complex three-dimensional substrate structures are required, conventional approaches have begun to reveal their limitations [66,67,139]. Therefore, at the sub-3 nm node, superior coating solutions still need to be explored.

Among the routes investigated to date, aerosol-spraying-based approaches and extrusion-spin-coating hybrid processes appear particularly promising, although they are intended to address different technical challenges. The former is primarily aimed at complex topographical surfaces, focusing on improving the coating coverage uniformity and local deposition controllability [140], while the latter attempts to compress the film thickness fluctuations and enhance the process predictability by reconstructing the most unstable initial spreading stage of spin-coating [67,141]. The core advantage of aerosol spraying is not merely the higher material utilization, but, rather, that its deposition method—based on droplets as the fundamental units—bypasses the reliance of conventional spin-coating on global centrifugal spreading, demonstrating its potential as an auxiliary step for spin-coating processes and serving as an effective supplementary means for local touch-up [142].

**Figure 2:**
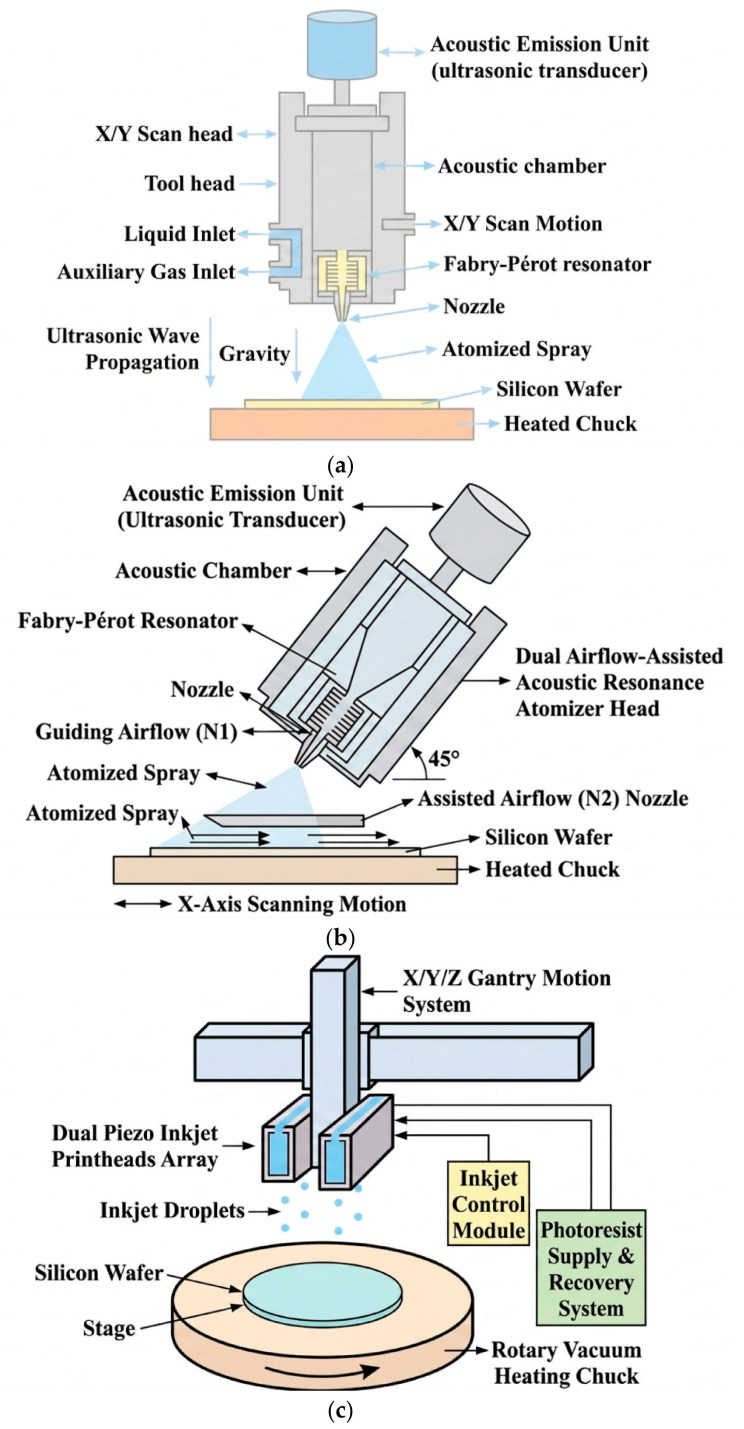
Principles of three photoresist coating technologies based on the aerosol spraying approach. (**a**) Schematic diagram of acoustic resonance spraying, where the photoresist is atomized into a uniform fine mist via a Fabry–Pérot resonant cavity. Adapted from [145]. (**b**) Schematic diagram of airflow-assisted acoustic spraying, where pinhole defects are suppressed and film uniformity is improved through an inclined nozzle and assisted airflow. Adapted from [143]. (**c**) Schematic diagram of spin-coupled inkjet printing, where large-area uniform film formation is achieved through staggered coating with multiple nozzles coupled with wafer rotation. Adapted from [144].

In recent years, techniques such as acoustic resonance atomization (see Figure 2a), airflow-assisted acoustic spraying (see Figure 2b), and spin-coupled inkjet printing (see Figure 2c) have emerged one after another. These are all advanced coating methods developed in recent years in the field of atomization and spraying. In advanced node scenarios, they can be better integrated with spin-coating processes for uniform film formation and used for localized touch-up coating. From the perspective of defect pathways, the value of such precision spraying strategies is mainly reflected in three aspects: they can suppress pinholes and local film rupture by stabilizing the droplet impact and spreading behavior [143]; mitigate the stitching region or local thickening issues by combining site-specific deposition with subsequent spin-coating homogenization [144]; and improve the deposition continuity and in-plane uniformity by enhancing the consistency of the droplet size and generation frequency [145]. However, it must be clearly stated that the advantages of spraying routes lie primarily in their coverage capability and local deposition flexibility, while their shortcomings fall precisely on the metrics most valued at advanced nodes, namely, ultrathin film thickness control, intra-wafer repeatability, and manufacturing robustness [2,146]. Therefore, they are better positioned as auxiliary homogenization or specific structure compensation means, rather than as a universal alternative for mainstream logic-layer EUV film formation.

**Figure 3:**
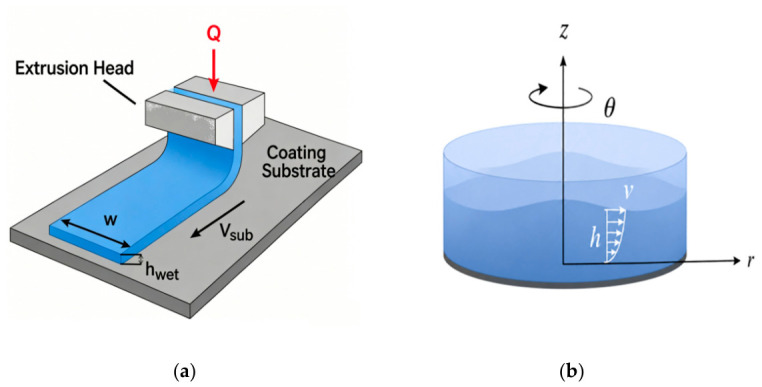
Principle and theoretical model of the extrusion-spin-coating process. (**a**) Schematic diagram of extrusion coating. Adapted from [141]. (**b**) Schematic diagram of film formation on a rotating disk based on the Emslie model. Adapted from [141].

Unlike spraying-based approaches, which attempt to alter the deposition mode itself, the extrusion-spin-coating hybrid process adopts a more process-compatible strategy and engineering-oriented strategy (Figure 3). Instead of replacing spin coating, this approach modifies the initial coating stage of the conventional process. The basic process flow involves first forming a more controllable pre-spread liquid layer using an extrusion head, where the photoresist is uniformly coated onto the wafer surface by an extrusion die. The wet-film thickness can be expressed as follows:

(8) $$h_{wet}=\frac{Q}{V_{sub}\omega }$$

Subsequently, high-speed rotation is employed to achieve film thinning, final thickness setting, and solvent evaporation curing [141]. The thickness evolution of the spin-coated layer over time can be described by the following:

(9) $$h=\frac{h_{0}}{{\left(1+\frac{t}{\tau }\right)}^{\frac{1}{2}}}$$

By bypassing the most unstable initial spreading stage of conventional spin coating, this strategy can reduce the thickness fluctuations at the source. Moreover, the fluid-dynamics-based thickness prediction models established for this process have improved the accuracy of the film-thickness estimation [147]. For conventional nodes or relatively thick-film applications, this route indeed offers considerable engineering value.

In summary, different film deposition technologies are not simply substitutes for traditional spin coating, but rather provide targeted solutions for different defect scenarios. However, from the perspective of sub-3 nm EUV manufacturing requirements, the evaluation of advanced film deposition technologies cannot focus solely on the coverage capability; it must also comprehensively consider the film thickness uniformity, surface roughness, defect density, and ultimate lithographic performance. Currently, spin coating remains dominant due to its mature process window and excellent on-wafer uniformity, while new spray coating and inkjet technologies serve primarily as important avenues of exploration for covering complex structures, local compensation, and future low-material-consumption processes. The future trend in advanced film deposition processes is not to completely replace spin coating, but, rather, to achieve a shift in photoresist film formation from “uniform deposition” toward “predictable defects and controllable performance” through the synergistic use of multiple deposition methods.The characteristics, advantages, limitations, and potential lithographic applications of these advanced coating approaches are summarized in Table 3.

However, when extended to the sub-3 nm technology node, the benefits of this engineering improvement may be rapidly offset by newly introduced error sources. Its main bottlenecks include at least two aspects. First, whether this method introduces new defect mechanisms at advanced technology nodes remains unclear, and systematic investigations and evaluation frameworks have yet to be established. Second, the uniformity control of this process in large-diameter wafer fabrication, as well as its process compatibility with existing semiconductor manufacturing systems, has not yet been fully validated or tested, and its adaptability and stability require further demonstration [141]. At advanced nodes, the requirements for photoresist thin films are no longer limited to meeting average thickness targets, but increasingly emphasize precise full-wafer uniformity and the strict suppression of local thickness variations [148]. As discussed earlier, the defect-generation mechanisms of photoresist films at these nodes differ significantly from those at previous generations. Therefore, at present, the extrusion-spin-coating hybrid route is more appropriately positioned as a structured enhancement of conventional spin coating rather than a mature mainstream replacement. Its applicability to the sub-3 nm node still requires extensive validation.

### 5.2. Development of Film Formation Prediction Models

As technology nodes continue to shrink, photoresist films are increasingly required to achieve ultrathin thicknesses, while the three-dimensional structures on wafer surfaces are becoming increasingly complex and process windows are progressively narrowing. Under these conditions, the traditional trial-and-error approach based primarily on empirical tuning is no longer sufficient for high-precision film-formation control [146]. Against this background, film-formation prediction models have evolved from early mechanistic analysis tools into key enabling technologies for process development and online control. In addition to reducing the experimental cost, accelerating the parameter screening, and defining the process windows, such models can also provide quantifiable state variables and physical prior constraints for virtual metrology, digital twins, and closed-loop process control [149].

The current mainstream spin-coating film formation models can be classified into three categories based on modeling dimensionality and fidelity: low-dimensional average models, low-dimensional extended models incorporating free boundary evolution, and high-fidelity numerical fluid models. These three categories represent different trade-offs among the computational cost, physical characterization accuracy, and engineering deployability on the production line [66,150].

The first category, represented by the Emslie model, the Bornside model, and their various derivative forms [66,147], takes the evolution of average film thickness as the core research object. Such models have a low computational overhead and are simple to implement, making them suitable for a rapid analysis of overall film thickness trends and preliminary process window screening—thus retaining practical engineering value to date [151]. However, these models adopt the lubrication approximation and assume that the film thickness varies only radially in one dimension or is uniformly distributed across the entire domain. They cannot accurately characterize free boundary movement, droplet spreading, coverage range changes, or the spatially non-uniform evolution of the film morphology during the evaporation and drying processes, resulting in inherent accuracy limitations [66,151].

To improve the representation of free-boundary evolution, one-dimensional spin-coating simulation models based on moving meshes have been proposed [152]. This approach employs moving mesh technology to numerically investigate the effects of the initial profile, dispensed volume, solvent vapor pressure, relative humidity, and initial viscosity on the geometry of the coated film [152]. Compared with traditional one-dimensional average models, the moving mesh approach offers a stronger characterization capability for spreading after dispensing and boundary evolution during subsequent evaporation and drying processes, while maintaining a relatively low computational cost. It is therefore more suitable for the preliminary process-window definition, parameter-sensitivity analysis, and rapid optimization for large-diameter wafers [66,152]. However, this type of model is still constructed based on the lubrication approximation and continuum theory. In nanoscale ultrathin film scenarios, intermolecular forces, interfacial wetting effects, and surface tension at the microscopic scale become dominant, substantially undermining the theoretical applicability of traditional continuum models [153].

In contrast, higher-dimensional numerical models offer advantages in physical fidelity and spatial resolution, but their application currently remains primarily focused on mechanistic analysis and offline simulation, still leaving a gap relative to the fab-level process optimization at advanced nodes. Their main limitations are twofold. First, most existing studies are still based on planar wafers and idealized boundary conditions, and therefore provide only a limited representation of complex three-dimensional patterned substrates. Second, the trade-off among predictive accuracy, parameter identifiability, and computational cost continues to constrain their direct use in high-precision process optimization. In addition, many numerical models still treat material constitutive behavior, evaporation kinetics, and interfacial conditions in a simplified manner, and therefore cannot fully reproduce actual film-formation behavior. Most reported studies also remain at the simulation stage, lack systematic benchmarking against production-line metrology data, and have not yet established a generalized parametric design framework suitable for high-speed rotation, low dispense volume, and ultrathin-film conditions [148].

More fundamentally, whether based on one-dimensional simplified formulations or on a high-fidelity numerical simulation, continuum-based models inevitably encounter common limitations when applied to ultrathin films, including idealized boundary assumptions and the inability to explicitly capture molecular-scale effects [66,153]. Therefore, a more worthwhile direction for further advancement is not simply to increase the geometric complexity or mesh density, but rather to develop multiscale models that explicitly couple interfacial wetting, evaporation kinetics, local material heterogeneity, and chemical reaction kinetics. Only by further combining these with data-driven approaches can a film formation simulation framework possessing both physical credibility and engineering computability be realized.

### 5.3. Optimization of Film Formation Environment and Interfacial Precision Control

As manufacturing enters the sub-3 nm advanced technology node, environmental conditions are no longer negligible secondary factors, but rather core variables that govern the film formation quality and determine the defect evolution trends [154]. The scope of environmental control has now expanded from traditional cleanroom-level temperature, humidity, and particle management to refined parameters such as the solvent vapor partial pressure, local temperature field, external force field, and interfacial microscopic states. In ultrathin film and high-density patterning manufacturing scenarios, fluctuations in environmental parameters are continuously amplified, and therefore must be incorporated into the main process closed-loop control framework.

**Figure 4:**
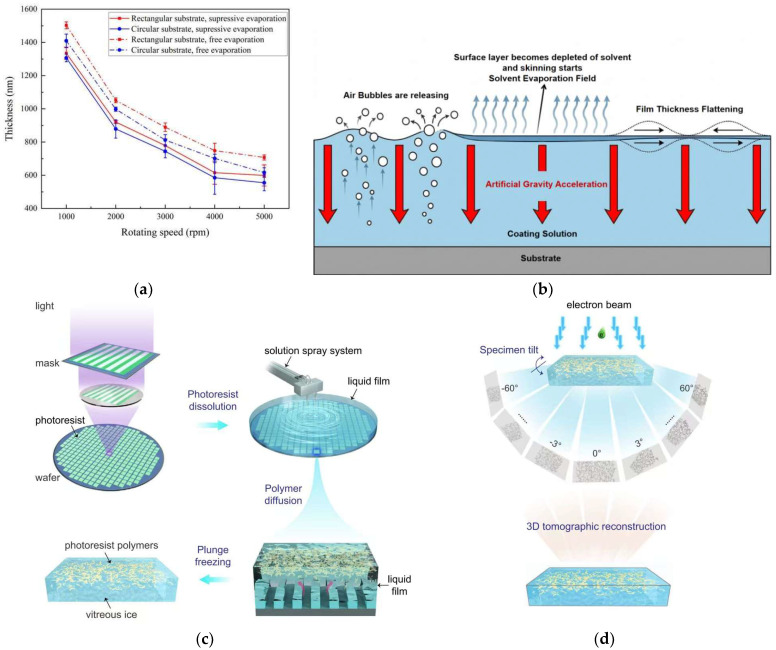
Environmental and interfacial regulation strategies for photoresist film formation at advanced nodes. (**a**) Comparison of spin-coated film thickness on rectangular/circular substrates under free evaporation and suppressed evaporation environments. Increasing the solvent vapor partial pressure in the chamber (suppressed evaporation) is a representative environmental regulation strategy for improving coating uniformity [151] ©2021, MDPI (CC BY 4.0). (**b**) Schematic diagram of the mechanism of artificial-gravity-driven bubble migration and expulsion under two-dimensional spin-coating conditions. Adapted from [159]. (**c**,**d**) Principle of in situ observation and three-dimensional reconstruction of liquid-phase photoresist polymers based on cryo-electron tomography [161] ©2025, Springer Nature (CC BY 4.0).

To address the issue of uneven evaporation during the spin-coating process described above, regulating the partial pressure of solvent vapor in the chamber is a key optimization strategy. This approach reduces the concentration gradient at the gas–liquid interface, allowing the solvent evaporation rate to become more consistent across the entire wafer, thereby effectively improving the overall film thickness uniformity and reducing the on-wafer thickness variations (see Figure 4a) [151,155]. The underlying mechanism is that a higher solvent partial pressure lowers the concentration gradient at the gas–liquid interface, allowing evaporation rates at different locations to become more consistent, thereby alleviating the problem of excessively rapid drying in edge regions due to greater exposure [156,157]. This approach is applicable to both complex substrates and large-diameter wafers, reducing defects caused by localized rapid drying. However, the effectiveness of such a regulation is jointly constrained by the photoresist composition, substrate topography, and chamber conditions, and thus requires calibration within specific process windows.

Bubble retention is an important factor affecting film densification, and artificial gravity field regulation serves as a targeted solution. By enhancing the effective gravitational acceleration, bubble ascent and removal can be accelerated, eliminating internal voids before film solidification and improving the overall film uniformity [158]. Both experimental and numerical studies have shown that a moderate increase in artificial gravity can promote bubble migration and expulsion, reduce internal voids and residual pores before solidification, and improve film densification and thickness uniformity (Figure 4b) [159]. However, the trade-off is equally evident: although a stronger artificial gravity may benefit internal densification, excessive loading can induce surface stress concentration, resulting in cracking, weakened adhesion, and local morphological instability [160]. Therefore, in practical applications, a balance must be struck between defect suppression and process safety.

Control of the development step has always been a challenge in lithography, as the process cannot be directly observed in real time, and traditional optimization relies on inferring process parameters backward from the final pattern results [74]. In recent years, the development of in situ and quasi-in situ microscopic observation techniques has provided new tools for studying dissolution kinetics. Among these, cryo-electron tomography enables the nanoscale in situ observation of photoresists in the liquid phase (see Figure 4c,d) [161]. This technique allows the quantitative extraction of key parameters such as swollen layer thickness, porosity, dissolution front roughness, and residual cluster size, establishing quantitative correlations between the process parameters and final yield. The technique can also be extended to scenarios such as wet cleaning and pre-/post-etching interfacial analysis, providing experimental support for full-process defect control.

In summary, the core value of environmental and interfacial precision control lies in transforming passively fluctuating external disturbances into quantifiable, modelable, and dynamically adjustable process variables. As relevant technologies continue to mature, film formation processes will evolve from single-parameter correction to a synergistic control mode encompassing evaporation kinetics, interfacial behavior, and microscopic defect evolution, ultimately achieving stable process windows.

### 5.4. Summary of This Chapter

Even with the continued advances in photoresist materials, it remains difficult to completely avoid film-formation defects introduced during processing. For this reason, targeted optimization at the process level remains indispensable. Representative directions include upgrading coating methods, improving film-formation prediction models, and implementing precise environmental and interfacial control, all of which play important roles in suppressing film-formation-related defects.

At the same time, the new defect modes emerging at the sub-3 nm node have already exceeded the explanatory and predictive capacity of traditional empirical knowledge. Process optimization alone is therefore unlikely to provide a complete solution. The introduction of intelligent methods to enhance optimization efficiency, accelerate defect identification, and strengthen process adaptability has become increasingly inevitable. Meanwhile, process innovation must also be coordinated with the aforementioned advances in material systems. Only through such a synergistic adaptation can the technical challenges of advanced-node manufacturing be addressed more effectively.

## 6. Defects Intelligent Regulation Systems for Mitigating Photoresist Film-Formation Defects

### 6.1. Principles for Applying Intelligent Strategies to Film-Formation Defects

As discussed in the preceding sections, photoresist film-formation-related defects at the sub-3 nm node exhibit new characteristics, while the effectiveness of traditional control measures is becoming increasingly constrained. Against this background, artificial intelligence (AI) is emerging as an important supplementary tool for defect monitoring and dynamic regulation.

However, the current application of AI in this field is not yet fully mature, which imposes certain requirements on its utilization. For specific defect objects, a verifiable mapping relationship from the data sources to control actions must be established. That is, different defects require different data, different models, different response speeds, and different control strategies. Only when this link is clear and verifiable can model outputs be truly transformed into executable process decisions [161].

Moreover, the various defect types that arise throughout the lithographic process differ significantly in both the formation mechanism and data representation. Consequently, intelligent regulation should be developed in a defect-oriented manner rather than through a single generalized model applied indiscriminately to all scenarios. Table 4 summarizes the technical framework of intelligent regulation for representative film-formation-related defects.

From the perspective of specific defect objects, film thickness non-uniformity and process drift typically exhibit strong continuity and temporal correlation, making them more suitable for time-series prediction or regression modeling frameworks [161], including methods such as LSTM, time-series regression, and ensemble learning [162,163]. The value of such models lies not merely in generating smoother thickness-prediction curves, but in using historical process data to determine when drift begins, how rapidly it evolves, and in which direction it develops, thereby enabling advance correction signals [171]. In contrast, issues such as edge bead rely more heavily on spatial distribution information and texture features, requiring the combination of in-line metrology and surface morphology imaging for rapid local early warning [164]. For defects such as pinholes and film breakage, image classification or segmentation networks are suitable for extracting features from microscopic images to achieve identification and localization [167,168]. However, because such defects are often extremely rare and strongly imbalanced in class distribution, models tend to learn only the characteristics of normal samples while overlooking true anomalies. In practice, this problem necessitates the use of data augmentation, resampling, or anomaly-detection strategies [165,166]. As for the systematic process drift caused by the combined effects of equipment, materials, and the environment, the causes are complex and accumulate over time. A single classification model is often insufficient; a more reasonable approach is to integrate intelligent models with advanced process control (APC) and digital twin systems, achieving closed-loop regulation through state estimation and feedback correction [149,169,170]. Thus, no single model can address all defects. The choice of method must first consider the defect type, data characteristics, and response speed, rather than chasing popularity or taking shortcuts.

Accordingly, the discussion of AI methods should not be limited to the model architecture or comparisons of the offline prediction accuracy. It is equally necessary to specify the input variables, output targets, response latency, deployment location, and evaluation metrics. Only when a clear correspondence is established between the model and a specific defect type, process scenario, and control requirement does intelligent regulation acquire real engineering significance [149,170]. Otherwise, so-called “smart manufacturing” remains merely a conceptual label, incapable of forming verifiable, executable, and accountable control capabilities on actual production lines.Representative AI-based approaches for photoresist defect prediction, detection, and process regulation, together with their input data, output objectives, and recent research progress, are summarized in Table 5.

### 6.2. Virtual Metrology and Digital Twin

Virtual metrology, digital twins, and advanced process control (APC) correspond, respectively, to the three levels of state perception, process characterization, and feedback regulation in the intelligentization of film formation processes. If virtual metrology primarily addresses the issue of “seeing the process state as early as possible,” then digital twins and APC further aim to address how to characterize the state evolution, predict the quality risks, and convert the prediction results into executable control actions [174,175].

The core of virtual metrology lies in using equipment sensor data, environmental parameters, process setpoints, and historical quality information to partially replace or predict in advance the offline metrology results, thereby enabling the rapid judgment of risks such as the film thickness drift and edge bead anomalies before physical metrology is completed [149,176]. Its main advantages include the rapid response, low cost, and ease of integration into in-line monitoring flows, making it well-suited to high-frequency, low-latency quality-monitoring scenarios. Under controlled conditions involving a single resist type, a single tool, and high-quality data, virtual metrology can generally capture mainstream quality-fluctuation trends effectively and provide useful functions in anomaly warning and parameter recommendation [176,177]. However, such methods are highly sensitive to the representativeness of the training data, sensor stability, and consistency of the metrology link. Once the equipment state drifts, batch fluctuations increase, or the resist type changes, the model accuracy and early warning reliability often degrade significantly [178].

In contrast, a digital twin is not merely a simple digital replica of the real equipment interface. Rather, it achieves dynamic simulation, state estimation, risk prediction, and decision support for manufacturing processes by constructing mapping relationships among equipment, process flow, material state, and product quality [169,174]. Unlike models that perform offline prediction based solely on historical data, digital twins emphasize virtual–real interaction and continuous updating. Their core lies not in “recreating a model,” but in integrating process perception, mechanistic understanding, and control feedback into a unified closed loop [174,179].

A typical workflow of a digital twin combined with APC can be summarized as follows: process data acquisition, state estimation, risk prediction, parameter correction recommendations, feedback execution, and result validation [175,180]. Within this workflow, state estimation maps multi-source sensor data into interpretable process state variables; risk prediction identifies issues such as film thickness drift, edge bead, or degraded repeatability; and the parameter correction module further outputs executable control strategies, such as adjustment schemes for spin-coating speed, acceleration, dispense volume, or temperature control parameters [175,177,180]. Only when the model output can actually act on subsequent process parameters, and its effects can be validated through the results of the next production run, does this process constitute closed-loop control in the strict sense. Otherwise, it essentially remains at the level of prediction, alarm, or decision support [175].

From a system perspective, the integration of a digital twin and APC transforms the intelligent regulation of film formation processes from mere result prediction into a closed-loop decision-making mechanism oriented toward state evolution and process optimization. Its value lies not only in characterizing the current process condition, but also in enabling the forward-looking assessment of future trends and the continuous iterative refinement of control strategies [174,179]. With a deeper integration of sensors, industrial communication protocols, mechanistic models, and data-driven algorithms, digital twins are expected to become a key hub connecting process monitoring, quality prediction, and online control, thereby supporting high-precision, low-variation, and adaptive regulation in advanced manufacturing [169,174].

However, it must be pointed out that not all defects are suitable for incorporation into a closed-loop control link. Only when a defect possesses observable precursor signals, an executable parameter correction path, and verifiable feedback results in subsequent batches does data intelligence truly constitute control. Otherwise, its role largely remains at the level of risk identification, anomaly screening, and the prioritization of disposal actions [175,181].

### 6.3. Limitations and Practical Challenges of Intelligent Methods

The principal bottlenecks of the current intelligent methods for photoresist film-formation-related defect control often arise not from the algorithms themselves, but from the mismatch among data distribution, physical constraints, detection latency, and process executability [182]. First, critical defect samples are scarce and labeling is costly. Defects such as stochastic bridging, local film rupture, and rare residue defects are intrinsically infrequent, while their identification often depends on experienced engineers and expensive inspection tools. As a result, the amount of labeled data available for model training is extremely limited, rendering conventional supervised-learning models fundamentally unstable in many cases [166,183]. Second, a model that works well on one tool or production line often fails on another. Variations in the spin-coating equipment status, airflow fields, substrate treatment, resist batches, and metrology tool differences can all lead to changes in data distribution. The decision rules learned by a model in its original environment often become directly invalid when transferred to a new environment [184]. Third, model interpretability remains insufficient [185,186]. Particularly when a model outputs a high-risk warning, if the result cannot be traced back to specific causes—such as temperature and humidity drift, viscosity changes, or bake temperature deviations—then, even if its statistical accuracy is high, it is difficult to be truly adopted by engineering teams [186]. Fourth, data sharing and joint modeling remain strongly constrained. In advanced manufacturing, formulations, process windows, and failure modes are typically protected by intellectual-property restrictions and confidentiality agreements, which makes large-scale cross-scenario collaborative modeling difficult to achieve [182]. Fifth, existing models generally have a weak adaptability to new defect modes. Most models are trained on historical data distributions. Once new resist types, new underlayer materials, new exposure conditions, or new developer formulations are introduced, bringing previously unseen failure mechanisms, the model is likely to degrade silently without any warning—a situation that is particularly dangerous in advanced node manufacturing [184,187].

A more feasible approach is to embed physical prior knowledge, causal sensitivity analysis, and uncertainty quantification into model training and deployment, enabling the model to provide not only prediction results but also confidence levels, key influencing factors, and actionable parameter adjustment recommendations [186,187,188]. It is particularly important to clarify that, for defects dominated by the inherent stochasticity of the material itself and lacking effective precursor observation signals, the functional boundary of intelligent models lies primarily in risk identification, tail constraint, and the prioritization of disposal actions, rather than in eliminating the generation mechanism of defects at the source.

### 6.4. Summary of This Chapter

Many defects emerging at the sub-3 nm node have exceeded the scope of traditional empirical knowledge. Therefore, introducing intelligent regulation methods to assist process optimization is becoming increasingly inevitable, helping to improve defect detection efficiency and capture minor anomalies more sensitively.

However, the current intelligent methods still face numerous challenges in photoresist defect control, such as the scarcity of defect samples and the susceptibility of processes to environmental disturbances. Consequently, at the present stage, intelligent methods are not yet capable of independently undertaking the task of defect discovery and resolution. They serve more as auxiliary means, working in conjunction with traditional process methods to identify defects and providing references and optimization suggestions for materials design through algorithmic analysis.

## 7. Synergistic Integration: A Material–Process–Intelligence Integrated Framework for Verifiable Defect Control

### 7.1. System-Level Challenges in Defect Control for Advanced Nodes

In sub-3 nm lithography, the generation and evolution of photoresist film-formation-related defects are governed by multivariate coupling and multistep propagation. Under such conditions, traditional single-parameter optimization is no longer sufficient to resolve the associated defect problems. As discussed in Section 3, Section 4 and Section 5, the current research progress and practical bottlenecks can be broadly categorized into three dimensions: photoresist materials, process control, and data-driven intelligent methods. However, these three technical domains are still largely developed in parallel and remain serially disconnected in practice. As a result, single-dimensional optimization can provide only a limited suppression of localized defects and cannot satisfy the stringent defect-control requirements of sub-3 nm lithography.

First, material improvement can only define the intrinsic lower bound of defect control, but cannot independently eliminate the extrinsic fluctuations introduced during subsequent steps—including spin-coating, baking, post-exposure bake, development, and etching—by the equipment state, environmental drift, and interfacial response variations [189,190]. Second, process parameter tuning can only marginally optimize the process window within a fixed photoresist system, but cannot reversely guide the targeted design of molecular structures, nor fundamentally mitigate stochastic defects caused by EUV photon fluctuations and interfacial non-uniformities. Third, intelligent tools such as machine learning and digital twins are highly dependent on high-quality, unbiased, and transferable datasets [149,170,182]. In the absence of constraints from material reaction kinetics, process physics, and causal mechanisms, these models can only fit correlations within the training data. When transferred to different tools or different photoresist batches, prediction failures are highly likely to occur, and it becomes difficult to generate physically interpretable and fab-implementable control solutions [187,188,191].

Accordingly, this section proposes a three-dimensional synergistic framework integrating materials, processes, and intelligence. By breaking down the barriers among these dimensions and establishing a bidirectional dynamic feedback network, the framework aims to transform defect control from isolated pointwise suppression into global and systematic governance.

### 7.2. Core Logic and Dimensional Positioning of the Three-Dimensional Synergistic Framework

**Figure 5:**
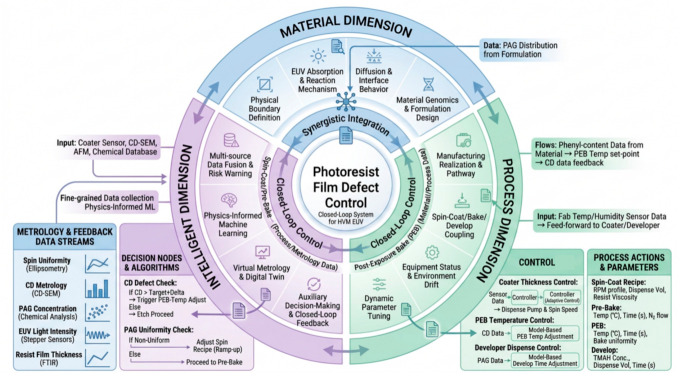
Schematic diagram of the material–process–intelligence three-dimensional synergistic regulation framework for photoresist film-formation-related defect control. This circular diagram intuitively illustrates the synergistic integration of materials design, process optimization, and intelligent closed-loop control, defines the core functional modules of each dimension and their mutual feedback mechanisms, and aims to address the fundamental challenges in photoresist film-formation-related defect control at the sub-3 nm node.

Based on the systematic drawbacks of merely single-dimensional optimization summarized above, this review builds upon previous research, focuses on the control of photoresist film-forming defects, and proposes a material–process–intelligence three-dimensional collaborative regulation framework targeted at governing photoresist film-formation defects (see Figure 5). Distinguished from existing studies that simply juxtapose the three types of technologies in a serial one-way optimization manner, this framework takes the complete causal evolution chain of defects as its core link, establishing a bidirectional closed-loop synergistic system in which “materials define physical boundaries—processes accomplish performance transformation—intelligence enables dynamic correction.”

#### 7.2.1. Core Functional Positioning of Each Dimension

Within the three-dimensional framework, the three entities do not operate independently. Rather, they each perform distinct functions while continuously constraining and dynamically empowering one another, collectively constituting a complete defect governance system. The core role of each dimension focuses on its value within the synergistic system, rather than merely reiterating the function of individual technologies.

In this framework, the materials dimension first defines the physical boundaries of system performance and the achievable lower limit of defects. The EUV absorption capability, reaction mechanism, diffusion behavior of acids or reactive intermediates, interfacial compatibility, film formation stability, and etch resistance of the photoresist collectively determine the lower bound of achievable defects and also predefine the adjustable range for subsequent processes [189,190].

The process dimension then determines whether the material potential can be translated into stable manufacturing results. Steps such as spin-coating, spraying, baking, post-exposure bake, development, cleaning, and etching constitute a continuously coupled process chain. Their control precision determines whether the intrinsic advantages of the material are preserved and amplified into a process window, or are offset by disturbances, drift, and interfacial instability during the cross-step transfer [192].

The intelligence dimension serves as a system-level feedback hub, collecting real-time data, sensing process states, performing virtual metrology, identifying abnormal conditions, and making online decisions. It converts deviations between actual process states and targets into interpretable, executable, and verifiable regulation instructions, thereby enabling dynamic correction and closed-loop optimization [149,169,170].

#### 7.2.2. Three-Dimensional Bidirectional Collaborative Operation Mechanism

The core innovation of this framework lies in abandoning the serial one-way optimization approach and establishing a global bidirectional dynamic feedback network—this is also the key distinction from traditional segmented R&D models. The three dimensions form a triple coupling relationship around the complete closed loop of defect generation, propagation, regulation, and iterative optimization:

Material–Process Bidirectional Constraint: The intrinsic physicochemical properties of the photoresist define the adjustable range of the supporting processes, thereby avoiding blind parameter traversal and trial-and-error approaches that deviate from the underlying physical mechanisms. Conversely, the adaptation requirements of complex three-dimensional substrates, multi-step disturbance characteristics, and mass-production stable process window constraints impose reverse requirements on key material design parameters—including viscosity, solvent volatility, acid diffusion length, and interfacial energy—during the molecular design stage, thereby reducing the trial-and-error costs of new material introduction into mass production [189,190,193].

Process–Intelligence Dynamic Adaptation: Intelligent systems perceive slow process drifts and dynamic defect evolution characteristics in real time, breaking through the limitations of fixed static process windows and outputting executable parameter correction instructions. Meanwhile, the mass-production sensor data, offline metrology results, and failure case data continuously accumulated at the process side are fed back to update intelligent models, enhancing their prediction robustness across different operating conditions and batches [149,169,170].

In semiconductor mass-production lines, full-wafer high-precision online inspection is not feasible. Traditional quality control methods can only employ sampling inspection, leaving information gaps regarding the true quality status of uninspected wafers. The stringent defect control standards of sub-3 nm advanced processes demand a more comprehensive coverage. Pre-trained virtual metrology models, without relying on physical inspection equipment, can perform the real-time inference of critical dimensions, film thickness, and defect risk levels for each individual wafer, achieving full-coverage wafer quality control. This not only reduces the inspection time and production costs, but also circumvents process regulation delays caused by the inspection latency.

Meanwhile, various types of production data on traditional production lines remain isolated from one another: the equipment degradation states, process operating parameters, and final wafer quality data are not interconnected in a closed loop. Once process anomalies occur, it becomes difficult to distinguish whether the root cause lies in the equipment aging, process parameter drift, or ambient environmental disturbances. Digital twin technology, by constructing coupled process–equipment simulation models, enables the precise traceability of anomaly sources and the localization of equipment drift, providing quantitative support for process parameter correction [180].

Virtual metrology and digital twin systems focus primarily on process anomaly perception, while intelligent optimization algorithms solve for optimal process parameters at a low computational cost and dynamically compensate for equipment performance degradation. This system, leveraging the massive production datasets generated by digital twin simulation models and virtual metrology, employs deep learning to mine historical process evolution patterns. Coupled with Bayesian optimization algorithms, it can substantially reduce the scale of physical test wafer experiments and rapidly converge to the optimal process window, addressing the traditional pain points of “high adjustment thresholds, lack of optimization basis, and significant tuning losses,” thereby significantly enhancing the process stability. On this basis, batch-level run-to-run (R2R) feedforward–feedback control enables automatic deviation correction for individual batches, stabilizing the baseline yield. Combined with iterative learning control (ILC), which continuously accumulates process optimization experience, long-term stable process operation can be further achieved [181,194,195].

However, the deployment of the above intelligent process systems is highly dependent on the accumulation of front-end full-process process data. The more complete the data sample dimensions and the larger the data volume, the more outstanding the prediction and optimization performance of the intelligent models. The two form an interdependent, synergistically reinforcing coupling relationship.

Intelligence–Material Iterative Optimization: Intelligent tools such as physics-informed neural networks (PINNs) and transfer learning assist in high-throughput material screening, moving beyond the limitations of manual trial and error to rapidly identify optimal photoresist formulations for different processes and substrate structures. The new physical property boundaries introduced by material iterations, in turn, recalibrate the built-in mechanistic constraints and optimization ranges of intelligent models, enabling the intelligent iterative upgrading of photoresist R&D.

Between intelligent algorithms and photoresist materials, a closed-loop synergistic relationship has been established in which “materials define the rules, AI learns the patterns, and AI deduces new materials”—the two complement rather than replace each other [11,188,196]. First, the molecular structure, formulation components, and physicochemical properties of the photoresist determine the inherent physical and chemical laws—including exposure reactions, acid diffusion, solvent evaporation, and dissolution kinetics—providing real physical constraint boundaries for the models [7,147]. Physics-informed neural networks (PINNs), grounded in these material mechanistic constraints, constrain the AI prediction range, preventing erroneous predictions that violate material principles in data-scarce, parameter-complex lithographic systems, thereby substantially improving the prediction stability across different materials and processes [188]. Second, transfer learning leverages the learning experience from mature photoresist materials to rapidly adapt to novel photoresist systems and new process conditions, addressing the challenge of insufficient data in new material development and enhancing material screening efficiency [197]. Second, transfer learning leverages the learning experience from mature photoresist materials to rapidly adapt to novel photoresist systems and new process conditions, addressing the challenge of insufficient data in new material development and enhancing the material screening efficiency [198,199]. Through this collaborative mode, material mechanisms constrain intelligent algorithms, while intelligent algorithms accelerate material iterative optimization, driving photoresist R&D from experience-based trial and error toward an intelligent, precision-oriented design.

The essence of three-dimensional synergy is a cross-scale, cross-disciplinary dynamic iterative system built around the defect governance objective, rather than a simple superposition of three types of technologies. Through multi-dimensional coupling, the blind spots of single-dimensional optimization are eliminated, achieving full-chain controllability from defect sources, through process execution, to mass-production endpoints.

#### 7.2.3. Operational Mechanism of the Three-Dimensional Synergistic System

Materials, processes, and intelligence constitute a full-process closed-loop synergistic system that spans the entire chain of photoresist R&D design, mass-production processing, online inspection, and parameter optimization. The three dimensions are deeply integrated and bidirectionally empowering, rather than merely engaged in simple pairwise coordination [200]. At the material design stage, the formulation and physicochemical properties can be directionally optimized by incorporating process characteristics and intelligent analysis results. Process debugging, in turn, is fully adapted to the physicochemical properties of the photoresist itself, and leverages intelligent inspection tools to rapidly identify defect issues in film formation, pattern transfer, and other steps. Intelligent algorithms then perform a dynamic correction of the process parameters, continuously iterating based on production metrology data [201,202]. The stable operation of the intelligent system also relies on the dual support of intrinsic material parameters and process operating data.

### 7.3. Engineering Obstacles, Failure Boundaries, and Evaluation Metrics

Although the material–process–intelligence synergistic system provides a comprehensive technical framework for photoresist film-formation-related defect control in advanced manufacturing, its practical deployment in mass production remains constrained by multiple factors, including the data infrastructure, metrology, workflow interfacing, control execution, and physical mechanisms [146]. First, the data standards across different production departments are not unified. Discrepancies in the data caliber, quality specifications, time stamps, lot traceability, and defect label definitions hinder the efficient fusion of data among materials R&D, process development, online inspection, failure analysis, and intelligent modeling [203]. Second, the core data—including material formulations, defect modes, process windows, and yield information—are often restricted by intellectual property rights, supply chain barriers, and confidentiality agreements, significantly constraining the model training, external validation, and cross-scenario adaptation capabilities [203,204]. Furthermore, the stable operation of a closed-loop control system relies on fast, accurate, and reliable metrology data. In high-takt manufacturing environments, issues such as inspection latency, insufficient sampling coverage, sensor drift, metrology tool offset, and missing data can all degrade the stability and reliability of the entire control system [149,170,176].

More critically, this synergistic framework cannot completely eliminate all photoresist film-formation-related defects [108]. Some defects are governed by the intrinsic stochasticity of the photoresist material itself and lack observable precursor signals. For such defects, intelligent models and closed-loop control can only provide a risk early warning, strict extreme-failure constraint, and optimized prioritization of disposal actions—they cannot eliminate the defect generation mechanism at the source. If a defect lacks observable precursor signals, lacks a feasible parameter optimization pathway, or if the optimization effect cannot be verified through subsequent metrology, it is not suitable for incorporation into the closed-loop control scope [181].

Therefore, the evaluation of this synergistic framework should not rely solely on superficial indicators—such as whether AI algorithms are introduced, whether a digital twin is established, or whether data links are interconnected—but, rather, focus on actual manufacturing outcomes and control effectiveness, establishing a quantitative evaluation system. Core evaluation metrics include the following: the defect density reduction, film thickness control error, LER/LWR improvement, residue and bridging occurrence rate, parameter drift recovery time, model false positive and false negative rates, cross-batch and cross-tool stability, model retraining frequency, control command execution success rate, and impact on production takt time [170,181]. Only when these metrics show sustained improvement under real production line conditions or standardized pilot-scale conditions—with the magnitude of improvement significantly exceeding the metrology error, sampling bias, and routine batch fluctuations, without compromising the production efficiency or process window stability—can the synergistic framework be considered to possess truly deployable and verifiable engineering value.

### 7.4. Summary of This Chapter

This chapter addresses the fundamental challenge in sub-3 nm advanced lithography processes, where single-dimensional material optimization, process parameter tuning, and intelligent modeling remain disconnected from one another and can only achieve localized defect suppression, without fundamentally resolving the underlying issues. To overcome this limitation, a material–process–intelligence integrated synergistic defect control framework has been constructed. The three-dimensional functional positioning has been clearly defined—materials delineate physical boundaries, processes convert manufacturing performance, and intelligence enables dynamic closed-loop correction—thereby breaking through the constraints of traditional point optimization and achieving an upgrade in photoresist film-formation-related defect control from localized mitigation to full-chain systematic governance. Furthermore, this chapter has analyzed the data barriers, metrology shortcomings, and physical failure boundaries that constrain framework deployment. Moving beyond formalistic evaluation criteria, a quantitative evaluation system oriented toward manufacturing outcomes has been established, clarifying the criteria for determining the genuine engineering application value of the synergistic framework. Collectively, this provides a systematic theoretical and technical foundation for stable, controllable, and deployable defect governance in advanced node lithography.

## 8. Challenges and Prospects

### 8.1. Key Challenges Currently Faced

Although materials design, process optimization, and intelligent control have each made progress, at the sub-3 nm and subsequent advanced nodes, these three capabilities have not yet formed a stable, transferable, and manufacturable unified system. More critically, material stochasticity, process disturbances, and data uncertainty are not independent of each other, but rather couple with and amplify each other during cross-step propagation [72,181].

At the materials level, the primary bottleneck for next-generation photoresists remains the difficulty of further compressing the intrinsic stochasticity. CAR systems are constrained by latent image blur caused by acid diffusion and the inherent resolution–sensitivity–roughness trade-off. Although non-CAR systems show potential in reaction localization, they often expose new engineering issues in cleanliness, interfacial compatibility, and pattern retention capability [4,72,138]. This means that improved resolution does not necessarily translate into a lower defect density or higher manufacturability [72]. Particularly under EUV and higher-NA exposure conditions, photoresist reactions have entered a regime co-governed by non-equilibrium photochemistry, stochastic effects, and other factors [2,49,205]. Issues at this length scale cannot be fundamentally resolved by simple formulation adjustments. Thus, while materials improvement can indeed enhance resolution, sensitivity, and etch resistance, it can hardly eliminate the rare but fatal stochastic defects. In advanced manufacturing, it is precisely these low-probability, high-damage rare failures that ultimately determine the chip yield [2,193,206].

At the process level, in high-aspect-ratio structures, complex three-dimensional topographies, and multi-step coupled flows, initial minor perturbations can be amplified stepwise during spin-coating, baking, development, etching, and cleaning processes, ultimately manifesting as film thickness non-uniformity, edge bead, local film breakage, development residue, or pattern transfer distortion [64,70,148,154,207]. Meanwhile, equipment aging, chamber contamination, environmental drift, and batch-to-batch variations continuously alter the position and width of the actual process window, making traditional control strategies that rely on fixed recipes, static windows, and empirical tuning unable to maintain the robustness, repeatability, and cross-batch consistency required for advanced manufacturing [175,181,208].

At the intelligence level, the key constraints on stable model deployment are not an insufficient variety of algorithms, but rather the scarcity of high-quality data, high labeling costs, weak cross-scenario transferability, and insufficient interpretability of results [149,203]. Defect samples in advanced manufacturing typically exhibit a low frequency, heterogeneity, and severe class imbalance, making it difficult for training data to cover the continuously evolving anomaly patterns in real production lines [165,166,168,209]. Moreover, significant data distribution shifts often exist across different tools, resist types, recipe windows, and process batches [178,184], causing models even with a high accuracy in a single scenario to fail rapidly when deployed across tools, resists, or batches. More importantly, if a model cannot trace anomaly determinations back to specific process variables, equipment states, or physical mechanisms, it can only provide statistical correlations without supporting executable engineering decisions in the fab. Such models, even with high offline metrics, struggle to gain the trust of process engineers [185,186].

At the synergy level, the truly difficult challenge is not advancing any single technology by another 5–10%, but rather how to organize materials, equipment, process, metrology, and yield information into a unified, feedback-capable, verifiable operating system [202,210]. High-value data are typically distributed across heterogeneous systems such as materials R&D, equipment control, process parameter databases, online metrology, yield analysis, and failure diagnosis. These systems commonly suffer from inconsistencies in data definitions, closed interfaces, incompatible standards, and intellectual property restrictions [200,202,210,211]. Consequently, although materials modification, process optimization, and intelligent modeling each advance, they still lack unified variable interfaces, metrology mappings, and feedback links. As a result, point improvements often only enhance localized metrics without translating into systemic gains in defect density, yield, or production line stability. Therefore, at advanced nodes, the true determinant of the upper limit is no longer whether any single technology is sufficiently “advanced,” but rather whether the entire manufacturing system can establish a closed-loop synergistic capability that connects materials design, process execution, online metrology, defect interpretation, and parameter feedback.

### 8.2. Key Future Trends

Looking ahead, defect control in film formation for advanced nodes will no longer be limited to the local optimization of individual process steps. Instead, it will evolve along the direction of the synergistic integration of materials, processes, and intelligence, and its objective will upgrade from localized defect correction to system-level optimization targeting defect density, process window, and yield stability [181,202,210,212].

Materials level: The future design of photoresists and related film-forming materials should not focus solely on improving the exposure speed and line width precision. Greater emphasis must be placed on the precise controllability of chemical reactions to ensure that reactions occur only in target regions, while also improving the film planarity and interfacial cleanliness. By suppressing the generation of stochastic micro-defects at the source and controlling the magnitude of line width fluctuations, the cumulative amplification of minor errors can be prevented, thereby ensuring the stability and reliability of photoresist products [9,72,138]. Consequently, the evaluation criteria for material optimization at advanced nodes will no longer focus only on the exposure speed and resolution metrics, but will also emphasize the defect control capability, pattern fidelity, compatibility with upstream and downstream processes, and process margin. This will fundamentally improve the film formation uniformity and stability, achieving a high precision and high consistency in pattern transfer.

Process level: With the continued development of gate-all-around (GAA), three-dimensional interconnects, and high-aspect-ratio structures [192,213], the film formation problem is no longer simply a matter of planar spreading, but has evolved into a coupled process influenced by multiple factors [70,71,149,154,157,207]. Future process optimization will focus on wetting regulation under complex topographies, local thickness homogenization, interfacial stabilization, and defect source suppression, thereby improving the conformal coverage of materials on three-dimensional structures and enhancing the stability of subsequent development and pattern transfer processes.

Intelligence level: As process complexity continues to increase, traditional control methods relying on empirical parameter tuning, fixed windows, and offline inspection can no longer support the real-time response, robustness, and traceability required for advanced manufacturing. In the future, in situ/quasi-in situ characterization, multiscale modeling, physics-informed intelligent control, and digital twins will accelerate their integration, driving film formation processes from experience-driven toward co-driven by mechanistic constraints and data feedback [7,149,174,187,188,212]. Among these, physics-informed models can explicitly embed constraints such as diffusion, heat transfer, reaction kinetics, and fluid behavior into the modeling process, thereby improving the prediction reliability under small-sample conditions and cross-scenario extrapolation capability [7,187,188]. Digital twins and virtual validation are expected to assume more core decision-support functions in defect early warning, parameter search, and closed-loop control, thereby shortening the process development cycles and reducing the trial-and-error costs [149,169,174,180,212,214].

In summary, achieving effective control of film formation defects in advanced process nodes will mainly focus on four directions: first, optimizing material systems to reduce material stochastic fluctuations and enhance action precision and localization; second, strengthening the adaptability between processes and structures to achieve more refined interfacial control; third, building a full-process in-line inspection and monitoring system to achieve real-time feedback and rapid response; and, fourth, integrating intelligent algorithms with physical mechanisms to form closed-loop intelligent regulation. The ultimate goal is to move beyond the experience-dependent mode of stepwise debugging and patchwork repair, and to systematically address film formation defects from a cross-scale, cross-process, cross-system global perspective.

### 8.3. Recommendations for Academia and Industry

For academia, subsequent research must first correct a common bias: single material performance or single pattern metrics can no longer be regarded as research endpoints. Instead, film formation stability, interfacial compatibility, development kinetics, defect statistical distribution, and manufacturing repeatability should be incorporated into a unified evaluation framework [3,72,138]. If research still takes ultimate resolution as the sole objective without addressing whether the film formation is stable, whether the interfaces are compatible, or whether the results can be reproduced across batches, its engineering value will be very limited. A more critical task is to transform the complex microscopic reactions, film formation processes, developer solution dynamics, and defect generation mechanisms in EUV lithography into observable variables, verifiable models, and comparable metrics, and to establish quantitative correlations between these quantities and equipment responses, process constraints, and manufacturing windows in actual fabs [74,79,134,161,215]. Meanwhile, explainable AI, physics-informed modeling, causal inference, and cross-scale coupling theory should become important interdisciplinary directions in this field. The value of these methods lies not in replacing experiments, but in narrowing the trial-and-error space, identifying high-sensitivity variables, and improving the extrapolation reliability of models under new resist types, new equipment, and new process conditions [186,187,188].

For industry, the most realistic and urgent task is not to continue stacking isolated algorithms, but rather to connect the data links among materials, equipment, process, metrology, and failure analysis, and to establish hierarchical feedback mechanisms around specific defect objects [181,202,210]. For example, when film thickness deviation occurs, it should be directly linked to virtual inspection and equipment state monitoring [149,214]; for edge bead issues, adjustments to the whole-wafer film thickness distribution, coating trajectory, and cleaning parameters must be coordinated [95,104]; for defects such as development residue and pattern bridging, all data from exposure, post-exposure bake, development, and final defect inspection must be correlated simultaneously; it is insufficient to build small models on individual process steps in isolation [2,74,189,215,216]. At advanced process nodes, truly effective optimization often relies not on more complex models, but on clearer defect standards, more stable data foundations, more reliable parameter correlations, and more stable closed-loop execution. Therefore, what industry needs to advance is not the continued escalation of localized automation, but rather system-level digital collaboration oriented toward material introduction, process upgrading, online metrology, and yield improvement, integrating these elements into a unified closed-loop operational framework [181,202,210,212]. Only on this basis can intelligent regulation transition from local pilot projects to fab-level system capability, demonstrating verifiable benefits in actual metrics such as the defect count, process stability, anomaly recovery speed, and production efficiency.

## 9. Conclusions

In summary, the formation of photoresist film-formation-related defects at the sub-3 nm node is not caused by a single factor, but is rather the result of dynamic evolution throughout the entire lithographic process flow. It is the product of the superposition of minor perturbations in front-end processes, the unique stochastic effects characteristic of advanced nodes, and the combined action of multi-dimensional factors including materials, processes, and environment. Moreover, at this node, stochasticity has become the fundamental core constraint determining pattern boundary stability, defect probability distribution, and process window range, which imposes far more stringent requirements on defect control than those at traditional nodes [2,72,206].

From the perspective of each control dimension, materials innovation serves as the fundamental core of defect control. Whether for traditional chemically amplified resist (CAR) systems or emerging non-chemically amplified resist (non-CAR) systems, their design and evaluation need to break through the limitations of single exposure performance and shift toward the comprehensive regulation of the full-process stability, balancing their own performance with environmental influences to reduce defects at the source. However, materials innovation has clear boundaries and cannot single-handedly eliminate subsequent failures caused by process disturbances, environmental drift, and cross-step amplification effects [4,72,79,128,131,132,138]. Process optimization serves as the key support for defect control. Through improvements in coating processes, upgrades in film formation prediction models, and the precision control of the environment and interface, it can effectively suppress film-formation-related defects. However, in the face of new types of defects at this node that exceed the scope of traditional empirical knowledge, relying solely on process optimization cannot fundamentally solve the problem. Intelligent regulation serves as an important auxiliary for defect control. Its introduction can improve the defect detection efficiency, capture minor anomalies, and provide references for materials design and process optimization. However, limited by issues such as scarce defect samples and environmental disturbances, it is not yet capable of independently undertaking the task of defect discovery and resolution, and can only serve as an auxiliary means in conjunction with traditional methods [4,11,169,203].

Based on the roles and limitations of the above dimensions, effective control of photoresist film-formation-related defects at the sub-3 nm node can no longer be achieved through single-dimensional improvements. It is necessary to construct an integrated “material–process–intelligence” synergistic regulation framework. In this framework, the materials dimension determines the intrinsic boundary and achievable lower limit of defect control; the process dimension determines the pathway for converting material properties into manufacturing outcomes and the degree of perturbation amplification; and the intelligence dimension provides technical support for multi-source data fusion, parameter feedback, and other functions. The three dimensions are not independent of each other, but rather interact and dynamically coordinate with one another—material characteristics constrain the adjustable range of processes, process parameters require real-time adjustment through intelligent monitoring, and intelligent technologies, combined with physical laws, assist in material screening and formulation optimization [13].

In the future, the key to achieving effective control of photoresist film-formation-related defects lies in establishing a complete closed loop covering materials design, process fabrication, inspection and analysis, model correction, and parameter re-optimization. For this closed loop to operate effectively, it requires observable precursor signals, adjustable process parameters, and verifiable improvement effects [11,181,212]. Meanwhile, for defects caused by material stochasticity that are difficult to monitor in advance, a reasonable objective should be risk identification and extreme failure constraint, rather than pursuing complete elimination. Through multi-dimensional synergistic efforts, reliable support with a low defect density, high robustness, and high manufacturability can be provided for sub-3 nm node patterning manufacturing.

## Figures and Tables

**Table 1:** Summary of core feature sizes for advanced processes at 3 nm and below [47,48].

Device Architecture	Process Node	Gate Length Lg	Contact-to-Gate Spacing CGP/CPP	Sub-Metal Half-Pitch	Transistor Density (100 million/cm^2^)
FinFET	3 nm (TSMC mass production)	12–14 nm	42–48 nm	22–23 nm	10–20
GAAFET	3 nm (Samsung mass production)	10–12 nm	26–38 nm	20–21 nm	30–80
GAAFET	2 nm (R&D)	8–10 nm	34–38 nm	16–18 nm	80–150
CFET	1 nm (long-term outlook)	10–12 nm	36–40 nm	14–16 nm	>300

**Table 2:** Performance characteristics (see text) of the most promising resist materials reviewed based on different design approaches.

Design Principle	Materials	Resolution	Sensitivity (Dose to Size)	Pattern Quality (LER-LWR)
CAR [88]	Polymeric	30 nm	<20 mJ/cm^2^	-
CAR [89]	Polymer-bound PAG	24 nm	14 mJ/cm^2^	5.3 nm
CAR [90]	Polymer-bound PAG—increased	16 nm	24 mJ/cm^2^	3 nm
CAR [91]	Polymeric	15 nm	25–30 mJ/cm^2^	6 nm
CAR [92]	Polymeric with different PAGs	13 nm	35.5 mJ/cm^2^	
CAR [93]	Polymeric	20 nm	31 mJ/cm^2^	-
CAR [94]	Polymeric	14 nm	43 mJ/cm^2^	5.8 nm
CAR [95]	Polymeric with Acid Amplifier (AA)	60 nm	1.9 mJ/cm^2^	7.9 nm
CAR—Multi-triggered resist [96,97,98,99]	Molecular	12.7 nm	53 mJ/cm^2^	4.2 nm
CAR [100]	Polymeric—main chain scission	20 nm	4 mJ/cm^2^	-
CAR [101]	Molecular	45 nm	10.3 mJ/cm^2^	-
CAR [102]	Molecular	45 nm	9.5 mJ/cm^2^	6.2 nm
CAR [103]	Molecular	26 nm	14.5 mJ/cm^2^	-
CAR [104]	Molecular	20 nm	40.5 mJ/cm^2^	3.2 nm
CAR [105]	Molecular	28 nm	22 mJ/cm^2^	3.7 nm
CAR [106,107]	Molecular	14 nm	36.1 mJ/cm^2^	3.26 nm
Non-CAR [108]	Polymeric	22 nm	78 mJ/cm^2^	<6 nm
Non-CAR [109]	Polymeric	20 nm	26.6 mJ/cm^2^	-
Non-CAR [110]	Polymeric	50 nm	52 mJ/cm^2^	4.1 nm
Inorganic [111,112]	Nanoparticles	26 nm	4.2 mJ/cm^2^	-
Inorganic [113]	Clusters	18 nm	350 mJ/cm^2^	-
Organometallic [114]	Molecular	35 nm	5.6 mJ/cm^2^	-
Organometallic [115]	Complexes	30 nm	90 mJ/cm^2^	5.5 nm
Metal [116]	-	17 nm	7 mJ/cm^2^	5.6 nm
Metal oxide [117]	-	13 nm	35 mJ/cm^2^	-
Metal–organic [118]	Clusters	13 nm	35 mJ/cm^2^	-
Metal [119]	Complexes	50 nm	53.5 mJ/cm^2^	-
Organohydrogen silsesquioxane [120]	Molecule	22 nm	65.4 mJ/cm^2^	1.4 nm
Metal oxide [121]	Clusters	25 nm	37 mJ/cm^2^	-

Reprinted from Ref [87], ©2020 MDPI (CC BY 4.0).

**Table 3:** Comparison of advanced atomization spraying and conventional rotational coating methods.

Film Deposition Technologies	Key Technical Features	Film Thickness Control	Surface Roughness	Uniformity	Photolithography Applications and Future Directions
Traditional Spin Coating	Utilizes centrifugal force to spread and thin the liquid film; this method has the highest level of industrial maturity.	Film thickness is adjusted with high precision by controlling rotational speed, viscosity, and solids content.	Surface quality is relatively high, but issues such as edge beads and unstable initial spreading remain.	Offers the best wafer-level uniformity and remains the mainstream EUV process.	It remains the primary method for depositing photoresist on advanced nodes, but its ability to cover complex structures is limited.
Acoustic Resonance Coating	Uses an acoustic field to control droplet size, enabling non-contact atomization and deposition.	Deposition volume is adjusted by controlling droplet size and spray parameters, gradually improving film thickness controllability.	Smaller droplets reduce localized accumulation, helping to minimize pinholes and surface irregularities.	Improves local deposition uniformity, making it suitable for covering complex structures.	It is suitable as a supplement to spin coating for special structures and localized defect repair.
Airflow-Assisted Acoustic Coating	Introduces an airflow to constrain the droplet’s trajectory based on acoustic atomization.	Film thickness is controlled by adjusting airflow velocity, nozzle angle, and deposition parameters.	Focus on improving defects such as pinholes and droplet aggregation.	Enhances film continuity and defect control capabilities.	It can improve photoresist film integrity, but its stability in mass production still needs further validation.
Spin-Coupled Inkjet	Combines inkjet-based metered liquid delivery with wafer rotation for spreading.	Digital thickness control is achieved by adjusting droplet volume and the printing path.	Reduce the traditional inkjet stitching pattern and improve film continuity.	Suitable for large-area uniform film deposition and the construction of multilayer structures.	It shows promise for applications requiring low material consumption, multilayer structures, and special use cases.

**Table 4:** Classification and technical strategies of the intelligent regulation system for photoresist film-formation-related defects.

Defect Type	Data Characteristic	Applicable AI/Intelligent Methods	Core Objective	Engineering Value
Film thickness non-uniformity/process drift	Time-series continuous, strong correlation, slow drift	LSTM, time-series regression, ensemble learning [161,162,163]	Predict drift start point, rate, and direction	Advance correction, reduce fluctuation
Edge bead	Spatial distribution, morphology texture, local anomaly	In-line metrology + image feature extraction [164]	Rapid local early warning, locate abnormal regions	Prevent edge morphology failure
Pinhole/film breakage/minor defects	Scarce samples, class imbalance [165,166]	Image classification/segmentation, anomaly detection, data augmentation [167,168]	Defect identification, precise localization	Reduce missed detection, minimize false positives
Systematic process drift (equipment/material/environment coupling)	Multi-source coupling, cumulative change, complex mechanism	Digital twin + APC + state estimation [149,169,170]	Closed-loop feedback, adaptive parameter correction	Stabilize process window, long-term controllability

**Table 5:** Recent advances in the application of artificial intelligence methods for defect control in advanced photoresists.

AI Methods	Challenges	Input Data	Output Objectives	Recent Research Findings
Machine-learning models such as LSTM and SVM	There is a complex interplay between photoresist formulations, exposure conditions, and process parameters, making traditional experimental optimization costly.	Photoresist composition, exposure conditions, experimental results, structural dimension data.	Predict CD and identify process windows.	Zhao et al. combined machine learning with electron beam lithography experiments to establish an LSTM prediction model and used SVM to screen for process conditions that meet dimensional requirements, thereby optimizing the lithography process [172].
Deep-learning-based visual inspection (YOLO, CNN, etc.)	EUV pattern defects are extremely small, making manual inspection using conventional SEM inefficient and resulting in an insufficient number of defect samples.	SEM images, synthetic defect data.	Defect classification and location identification.	Shinde et al. trained a YOLOv8 model using synthetic SEM data to detect “bridge” and “break” defects in lithographic patterns, achieving a model mAP of approximately 96% [173].
Virtual measurement models	In-line film thickness measurement is costly and has a long feedback cycle.	Equipment sensor data, environmental parameters, historical quality data.	Film thickness and defect risk prediction.	A mapping relationship between process parameters and quality metrics was established, enabling state prediction without the need for real-time physical measurements.
Digital twin + APC	Process drift is caused by a combination of equipment, material, and environmental factors, making it difficult to control using a single model.	Equipment status, process data, material status.	Parameter tuning strategies and closed-loop control.	Through virtual-to-real mapping and feedback control, process state prediction and dynamic parameter correction were achieved.

## Data Availability

This is a literature review, and no new experimental data were generated in this study, so data availability is not applicable.

## Abbreviations

The following abbreviations are used in this manuscript:

AFM	Atomic Force Microscope
AI	Artificial Intelligence
APC	Advanced Process Control
BCL	Block Copolymer Lithography
BEUV	Beyond Extreme Ultraviolet
CAR	Chemically Amplified Resist
CFET	Complementary Field-Effect Transistor
CD	Critical Dimension
CFD	Computational Fluid Dynamics
cryo-ET	Cryo-electron Tomography
DML	Digital Maskless Lithography
DPN	Dip-pen Nanolithography
DT	Digital Twin
DOP	Degree of Planarization
DUV	Deep Ultraviolet
EBL	Electron Beam Lithography
EHL	Electrohydrodynamic Lithography
EUV	Extreme Ultraviolet
EUVL	Extreme Ultraviolet Lithography
Fab	Semiconductor Fabrication Facility
FEL	Free-Electron Laser
FET	Field-Effect Transistor
GAA	Gate-All-Around
High-NA	High Numerical Aperture
Hyper-NA	Hyper-Numerical Aperture
HSQ	Hydrogen Silsesquioxane
HVM	High-Volume Manufacturing
IL	Interference Lithography
ILC	Iterative Learning Control
IML	Immersion Lithography
IoT	Internet of Things
LPP	Laser-Produced Plasma
LER	Line Edge Roughness
LSTM	Long Short-Term Memory
LWR	Line Width Roughness
MBCFET	Multi-Bridge Channel Field-Effect Transistor
MBML	Multi-Beam Maskless Lithography
MEMS	Micro-Electromechanical Systems
MG	Molecular Glass
MOR	Metal Oxide Resist
MTR	Multi-Triggered Resist
NA	Numerical Aperture
NIL	Nanoimprint Lithography
non-CAR	Non-chemically Amplified Resist
NSL	Nanosphere Lithography
PAG	Photoacid Generator
PDMS	Polydimethylsiloxane
PEB	Post-Exposure Bake
PINN	Physics-Informed Neural Network
PMGI	Polymethylglutarimide
PMMA	Poly(methyl methacrylate)
PR	Photoresist
PS	Polystyrene
PSL	Plasmonic Lithography
R2R	Run-to-Run
rpm	Revolutions per minute
SEM	Scanning Electron Microscope
SERS	Surface-Enhanced Raman Scattering
SL	Soft Lithography
SRAM	Static Random Access Memory
SPIE	International Society for Optics and Photonics
UV	Ultraviolet
VM	Virtual Metrology
XRL	X-Ray Lithography
TMAH	Tetramethylammonium Hydroxide

## References

[1] Patsis G., Gogolides E., Werden K.V. (2005). Effects of photoresist polymer molecular weight and acid-diffusion on line-edge roughness. Jpn. J. Appl. Phys..

[2] De Bisschop P., Hendrickx E. (2018). Stochastic effects in EUV lithography. Proceedings of the Extreme Ultraviolet (EUV) Lithography IX.

[3] Wang X., He J., Wei J., Zhu H. (2024). Stochastics in EUV Lithography and Recent Research Status. Chin. J. Lasers.

[4] Ghosh S., Pradeep C.P., Sharma S.K., Reddy P.G., Pal S.P., Gonsalves K.E. (2016). Recent advances in non-chemically amplified photoresists for next generation IC technology. RSC Adv..

[5] Scriven L. (1988). Physics and applications of dip coating and spin coating. MRS Online Proc. Libr..

[6] Arpitha V., Pani A.K. (2022). Machine learning approaches for fault detection in semiconductor manufacturing process: A critical review of recent applications and future perspectives. Chem. Biochem. Eng. Q..

[7] Karniadakis G.E., Kevrekidis I.G., Lu L., Perdikaris P., Wang S., Yang L. (2021). Physics-informed machine learning. Nat. Rev. Phys..

[8] Kazazis D., Santaclara J.G., van Schoot J., Mochi I., Ekinci Y. (2024). Extreme ultraviolet lithography. Nat. Rev. Methods Primers.

[9] Li M., Aqad E. (2025). Key Challenges and Opportunities for Advanced Extreme Ultraviolet Lithography Photoresist Materials. Adv. Funct. Mater..

[10] Kanarik K.J., Osowiecki W.T., Lu Y., Talukder D., Roschewsky N., Park S.N., Kamon M., Fried D.M., Gottscho R.A. (2023). Human–machine collaboration for improving semiconductor process development. Nature.

[11] Butler K.T., Davies D.W., Cartwright H., Isayev O., Walsh A. (2018). Machine learning for molecular and materials science. Nature.

[12] Xia J., Zhang Y., Jiang B. (2025). The evolution of machine learning potentials for molecules, reactions and materials. Chem. Soc. Rev..

[13] Zheng Y., Xu H., Li Z., Li L., Yu Y., Jiang P., Shi Y., Zhang J., Huang Y., Luo Q. (2025). Artificial Intelligence-Driven Approaches in Semiconductor Research. Adv. Mater..

[14] Fu N., Liu Y., Ma X., Chen Z. (2019). EUV lithography: State-of-the-art review. J. Microelectron. Manuf..

[15] Klomp P. (2025). 0.33 NA EUV systems for high-volume manufacturing. Proceedings of the Optical and EUV Nanolithography XXXVIII.

[16] Maas R., Dhaeze P., Teeuwisse F., Kwon O.-S., Salmaso G., Klomp P., Meijerink R., van Es R., Lavrijssen P., van de Kerkhof M. (2025). 0.33 NA EUV Systems for High-Volume Manufacturing. Proc. SPIE 13686, International Conference on Extreme Ultraviolet Lithography 2025.

[17] Basu P., Verma J., Abhinav V., Ratnesh R.K., Singla Y.K., Kumar V. (2025). Advancements in lithography techniques and emerging molecular strategies for nanostructure fabrication. Int. J. Mol. Sci..

[18] Van Schoot J., van Ballegoij R., Butler H., van Setten E., Schiffelers G., Bouman W., van Gorp S., Umstadter K., Zimmermann J., Golde D. (2025). Next step in Moore’s law: High NA EUV system overview and first imaging and overlay performance. J. Micro/Nanopattern. Mater. Metrol..

[19] Levinson H. (2025). The era of high-NA EUV lithography has arrived!. Jpn. J. Appl. Phys..

[20] Lee I., Franke J.-H., Philipsen V., Ronse K., De Gendt S., Hendrickx E. (2025). Study on next-generation EUV lithography technology: Hyper NA, the highest potential for practical implementation. ACS Appl. Mater. Interfaces.

[21] Benk M., Miyakawa R., Chao W., La Fontaine B. (2025). Mask-side Hyper-NA EUV imaging on the SHARP microscope. J. Micro/Nanopattern. Mater. Metrol..

[22] De Bisschop P. (2017). Stochastic effects in EUV lithography: Random, local CD variability, and printing failures. J. Micro/Nanolithogr. MEMS MOEMS.

[23] De Bisschop P. (2018). Stochastic printing failures in extreme ultraviolet lithography. J. Micro/Nanolithograp. MEMS MOEMS.

[24] Fukuda H. (2020). Cascade and cluster of correlated reactions as causes of stochastic defects in extreme ultraviolet lithography. J. Micro/Nanolithogr. MEMS MOEMS.

[25] Fomenkov I., Brandt D., Ershov A., Schafgans A., Tao Y., Vaschenko G., Rokitski S., Kats M., Vargas M., Purvis M. (2017). Light sources for high-volume manufacturing EUV lithography: Technology, performance, and power scaling. Adv. Opt. Technol..

[26] Fujimoto J., Hori T., Yanagida T., Mizoguchi H. (2012). Development of Laser-Produced Tin Plasma-Based EUV Light Source Technology for HVM EUV Lithography. Phys. Res. Int..

[27] Zhu Q., Schafgans A.A., Stewart J., Rich S., Purvis M., Wang H., Hummler K., LaForge A., Mayer P., Urone D. (2025). Evolution of EUV light source architecture for continued advancements in EUV high volume manufacturing. Proceedings of the Optical and EUV Nanolithography XXXVIII.

[28] Decking W., Abeghyan S., Abramian P., Abramsky A., Aguirre A., Albrecht C., Alou P., Altarelli M., Altmann P., Amyan K. (2020). A MHz-repetition-rate hard X-ray free-electron laser driven by a superconducting linear accelerator. Nat. Photonics.

[29] Kawata H., Nakamura N., Sakai H., Kato R., Hajima R. (2022). High power light source for future extreme ultraviolet lithography based on energy-recovery linac free-electron laser. J. Micro/Nanopattern. Mater. Metrol..

[30] Kumar A., Banerjee D. (2026). Review of Evolution and Advances in Photolithography and Nanopatterning Using Free-Electron Lasers. ASME Open J. Eng..

[31] Nakamura N., Kato R., Sakai H., Tsuchiya K., Tanimoto Y., Honda Y., Shimada M., Yamamoto M., Tanikawa T., Tanaka O. (2024). Research and development of the EUV-FEL light source for lithography. Proceedings of the Photomask Japan 2024: XXX Symposium on Photomask and Next-Generation Lithography Mask Technology.

[32] Gubarev V., Krivokorytov M., Stepanov L., Matiukhin N., Ivanov V., Antziferov P., Koshelev K. (2025). Recombination mode of extreme ultraviolet radiation in a lithium laser-produced plasma. J. Appl. Phys..

[33] Guseva V., Nechay A., Perekalov A., Salashchenko N., Chkhalo N. (2023). Investigation of emission spectra of plasma generated by laser pulses on Xe gas-jet targets. Appl. Physics. B Lasers Opt..

[34] Wen Z., Xie Z., Zhang Q., Wang S., Pu Z., Song X., Dou Y., Li B., Pan Q., Chen F. (2025). Enhancement of spectral performance in gadolinium-based BEUV sources by supplying pre-formed plasma with cavity-confined targets. Opt. Express.

[35] Nakamura N., Kato R., Sakai H., Tsuchiya K., Tanimoto Y., Honda Y., Miyajima T., Shimada M., Tanikawa T., Tanaka O.A. (2023). High-power EUV free-electron laser for future lithography. Jpn. J. Appl. Phys..

[36] Uzoma P.C., Shabbir S., Hu H., Okonkwo P.C., Penkov O.V. (2021). Multilayer reflective coatings for BEUV lithography: A review. Nanomaterials.

[37] Mojarad N., Gobrecht J., Ekinci Y. (2015). Beyond EUV lithography: A comparative study of efficient photoresists’ performance. Sci. Rep..

[38] Wu S.-Y., Chang C., Chiang M., Lin C., Liaw J., Cheng J., Yeh J., Chen H., Chang S., Lai K. (2022). A 3nm CMOS FinFlex™ platform technology with enhanced power efficiency and performance for mobile SoC and high performance computing applications. Proceedings of the 2022 International Electron Devices Meeting (IEDM).

[39] Bae G., Bae D.-I., Kang M., Hwang S., Kim S., Seo B., Kwon T., Lee T., Moon C., Choi Y. (2018). 3nm GAA technology featuring multi-bridge-channel FET for low power and high performance applications. Proceedings of the 2018 IEEE International Electron Devices Meeting (IEDM).

[40] Kim S., Park H., Choi E., Kim Y.H., Kim D., Shim H., Chung S., Jung P. (2023). Reliability assessment of 3nm GAA logic technology featuring multi-bridge-channel FETs. Proceedings of the 2023 IEEE International Reliability Physics Symposium (IRPS).

[41] Sinha A., Choudhary N. (2025). Gate-All-Around Transistors at 3nm: Device Physics, Fabrication Challenges, and Beyond FinFET Scaling. Res. Arch. Rising Sch..

[42] Mukesh S., Zhang J. (2022). A review of the gate-all-around nanosheet FET process opportunities. Electronics.

[43] Loubet N., Hook T., Montanini P., Yeung C.-W., Kanakasabapathy S., Guillom M., Yamashita T., Zhang J., Miao X., Wang J. (2017). Stacked nanosheet gate-all-around transistor to enable scaling beyond FinFET. Proceedings of the 2017 Symposium on VLSI Technology.

[44] Noh C., Han C., Won S.M., Shin C. (2022). Vertical gate-all-around device architecture to improve the device performance for sub-5-nm technology. Micromachines.

[45] Li S., Luo Y., Xu H., Huo J., Di Z., Li Y., Zhang Q., Yin H., Wu Z. (2023). Vertically stacked nanosheet number optimization strategy for complementary FET (CFET) scaling beyond 2 nm. IEEE Trans. Electron Devices.

[46] Ajayan J., Sreenivasulu V.B., Dwivedi A.K., Tayal S. (2025). Complementary Field Effect Transistor (CFET) for the 2-nm Technology Node: A Review From Device to Circuit Perspectives. IEEE J. Electron Devices Soc..

[47] Zhang Q., Zhang Y., Luo Y., Yin H. (2024). New structure transistors for advanced technology node CMOS ICs. Natl. Sci. Rev..

[48] Das U.K., Bhattacharyya T.K. (2020). Opportunities in device scaling for 3-nm node and beyond: FinFET versus GAA-FET versus UFET. IEEE Trans. Electron Devices.

[49] Brainard R.L., Trefonas P., Lammers J.H., Cutler C.A., Mackevich J.F., Trefonas A., Robertson S.A. (2004). Shot noise, LER, and quantum efficiency of EUV photoresists. Proceedings of the Emerging Lithographic Technologies VIII.

[50] Pret A.V., Graves T., Blankenship D., Bai K., Robertson S., De Bisschop P., Biafore J.J. (2018). Comparative stochastic process variation bands for N7, N5, and N3 at EUV. Proceedings of the Extreme Ultraviolet (EUV) Lithography IX.

[51] Libeert G., Franke J.-H., Leitao S., Davydova N., Ramachandran P.A., Varghese S.S.K., Philipsen V. (2025). Depth-of-focus enhancement in high-numerical aperture EUV lithography by source and mask optimization. Proceedings of the Photomask Technology 2025.

[52] Levinson H.J. (2022). High-NA EUV lithography: Current status and outlook for the future. Jpn. J. Appl. Phys..

[53] Ren H., Gronheid R., Gao X., Tang W., Cheng G., Chen Z., Zafar K., Gröger P., Bald H., Beaufort G.D.L. (2026). Wafer topography impact on focus and defectivity with enhanced method for EUV focus metrology. J. Micro/Nanopattern. Mater. Metrol..

[54] Seshadri I., Miller E., Church J., Chu A., Zhang J., Greene A., Frougier J., Li T., Cabrera Y., Kenath G. (2023). Scaling opportunities for Gate-All-Around: A patterning perspective. Proceedings of the 2023 International Electron Devices Meeting (IEDM), San Francisco, CA, USA, 9–13 December 2023.

[55] Goldfarb D., Chen Z., Cabrera Y., Petrillo K., Lee J., Meher W., Gu K., Matham S.K., Penny C., Rankin J. (2026). Exploring the Limits of Contact Hole Patterning with High-NA EUV Lithography. Proc. SPIE 13983, Advances in Patterning Materials and Processes XLIII.

[56] Rafael-Naab B.D., Park J.K., Aqad E., Cen Y., Coley S.M., Zacharias A.O., Cheng L., Cui L., Hoelzel C.A., Choi K. (2025). The impact of polymer compositional homogeneity on lithography and defects. Proceedings of the Advanced Lithography.

[57] Hosaka Y., Oyama T.G., Oshima A., Enomoto S., Washio M., Tagawa S. (2013). Pulse Radiolysis Study on a Highly Sensitive Chlorinated Resist ZEP520A. J. Photopolym. Sci. Technol..

[58] Kostko O., Im H., Kaplan S., Oh D. (2026). Deciphering EUV-induced chemistry in photoresists through in situ and ex situ characterization techniques. Proceedings of the Advanced Lithography.

[59] Tanenbaum D., Lo C., Isaacson M., Craighead H., Rooks M., Lee K., Huang W., Chang T. (1996). High resolution electron beam lithography using ZEP-520 and KRS resists at low voltage. J. Vac. Sci. Technol. B Microelectron. Nanometer Struct..

[60] Dam T., Jamieson A., Lu M., Baik K.H. (2006). PAB and PEB temperature gradient methodology for CAR optimization. Proc. SPIE.

[61] Xiang D., He L., Qu D., Mou P., Duan G. (2012). Development of coating of photoresist in semiconductor manufacturing. China Mech. Eng..

[62] Wu T., Zhu F., Wang Q. (2020). The Global Variation of Photoresist Topography and CD Uniformity due to Local High Step. Proceedings of the 2020 IEEE International Conference on Artificial Intelligence and Computer Applications (ICAICA).

[63] Ipatova A., Smirnov K.V., Mogilevskiy E.I. (2021). Steady circular hydraulic jump on a rotating disk. J. Fluid Mech..

[64] Mouhamad Y., Mokarian-Tabari P., Clarke N., Jones R.A.L., Geoghegan M. (2014). Dynamics of polymer film formation during spin coating. J. Appl. Phys..

[65] Kutchoukov V., Mollinger J., Bossche A. (2000). New photoresist coating method for 3-D structured wafers. Sens. Actuators A Phys..

[66] Bornside D.E., Macosko C.W., Scriven L.E. (1989). Spin coating: One-dimensional model. J. Appl. Phys..

[67] Meyerhofer D. (1978). Characteristics of resist films produced by spinning. J. Appl. Phys..

[68] Ho W.K., Tay A., Chen M., Fu J., Lu H., Shan X. (2007). Critical Dimension Uniformity Via Real-Time Photoresist Thickness Control. IEEE Trans. Semicond. Manuf..

[69] Cooper K.A., Hamel C., Whitney B., Weilermann K., Kramer K.-J., Zhao Y., Gentile H. (2007). Conformal Photoresist Coatings for High Aspect Ratio Features. Wafer-Level Packaging Symposium.

[70] Arscott S. (2020). The limits of edge bead planarization and surface levelling in spin-coated liquid films. J. Micromech. Microeng..

[71] Zhang Y.Y., D’Ambra C.A., Katsumata R., Burns R.L., Somervell M.H., Segalman R.A., Hawker C.J., Bates C.M. (2019). Rapid and Selective Deposition of Patterned Thin Films on Heterogeneous Substrates via Spin Coating. Acs Appl. Mater. Interfaces.

[72] Wang X., Tao P., Wang Q., Zhao R., Liu T., Hu Y., Hu Z., Wang Y., Wang J., Tang Y. (2023). Trends in photoresist materials for extreme ultraviolet lithography: A review. Mater. Today.

[73] Kim T.-Y., Kang I.-H., Park J., Kim M., Oh H.-K., Hur S.-M. (2023). Coarse-Grained Modeling of EUV Patterning Process Reflecting Photochemical Reactions and Chain Conformations. Polymers.

[74] Itani T., Santillan J.J. (2010). Dissolution Behavior of Photoresists: An In-situ Analysis. J. Photopolym. Sci. Technol..

[75] Wu B.Q. (2006). Photomask plasma etching: A review. J. Vac. Sci. Technol. B.

[76] Ito H., Willson C.G. (1983). Chemical amplification in the design of dry developing resist materials. Polym. Eng. Sci..

[77] Mack C.A., Biafore J.J., Smith M.D. (2011). Stochastic Acid-Based Quenching in Chemically Amplified Photoresists: A Simulation Study.

[78] Fukuda H. (2022). Stochasticity in extreme-ultraviolet lithography predicted by principal component analysis of Monte Carlo simulated event distributions in resist films. J. Appl. Phys..

[79] Wang Z.L., Du H., Xin H.S., Xue J., Zhang J.H., Li H.Y. (2024). Microscopic Mechanisms of Reaction-Coupled Acid Diffusion in Chemically Amplified Photoresists. Chem. Mater..

[80] Philippou A., Mülders T., Schoell E. (2007). Impact of photoresist composition and polymer chain length on line edge roughness probed with a stochastic simulator. J. Micro-Nanolithogr. MEMS MOEMS.

[81] Miyoshi H., Taniguchi J. (2015). Fabrication of a high-resolution mask by using variable-shaped electron beam lithography with a non-chemically amplified resist and a post-exposure bake. Microelectron. Eng..

[82] Wu B.Q., Kumar A. (2007). Plasma etch method for extreme ultraviolet lithography photomask. Appl. Phys. Lett..

[83] Auciello O., Gras-Martí A., Valles-Abarca J.A., Flamm D.L. (2012). Plasma-Surface Interactions and Processing of Materials.

[84] Miura T., Kekura M., Horibe H., Yamamoto M. (2008). Photo-resist removal using highly concentrated ozone gas—Removal characteristics of various resists. J. Photopolym. Sci. Technol..

[85] Naulleau P., Anderson C., Chao W., Bhattarai S., Neureuther A. (2016). Stochastics and EUV patterning in the 1x-nm regime. J. Photopolym. Sci. Technol..

[86] Han G., Hao Y. (2021). Design technology co-optimization towards sub-3 nm technology nodes. J. Semicond..

[87] Manouras T., Argitis P. (2020). High Sensitivity Resists for EUV Lithography: A Review of Material Design Strategies and Performance Results. Nanomaterials.

[88] Fedynyshyn T., Goodman R., Roberts J. (2008). Polymer matrix effects on acid generation. Proc. SPIE—Int. Soc. Opt. Eng..

[89] Tamaoki H., Tarutani S., Tsubaki H., Takahashi T., Inoue N., Tsuchihashi T., Takizawa H., Takahashi H. (2011). Characterizing Polymer Bound PAG-Type EUV Resist. Proc. SPIE 7972, Advances in Resist Materials and Processing Technology XXVIII.

[90] Tarutani S., Tsubaki H., Takizawa H., Goto T. (2012). EUV Resist Materials for 16 nm And below Half Pitch Applications. J. Photopolym. Sci. Technol..

[91] Tsubaki H., Tarutani S., Inoue N., Takizawa H., Goto T. (2013). EUV Resist Materials Design for 15nm Half Pitch and Below. Proc. SPIE 8679, Extreme Ultraviolet (EUV) Lithography IV.

[92] Fujiwara K. (2017). Novel EUV resist development for sub-14 nm half pitch. Proceedings of the 2017 China Semiconductor Technology International Conference (CSTIC), Shanghai, China, 12–13 March 2017.

[93] Thackeray J., Nassar R., Brainard R., Goldfarb D., Wallow T., Wei Y., Mackey J., Naulleau P., Pierson B., Solak H. (2007). Chemically amplified resists resolving 25 nm 1:1 line:Space features with EUV lithography. Proc. SPIE—Int. Soc. Opt. Eng..

[94] Fujii T., Matsumaru S., Yamada T., Komuro Y., Kawana D., Ohmori K. (2016). Patterning Performance of Chemically Amplified Resist in EUV Lithography. Proc. SPIE 9776, Extreme Ultraviolet (EUV) Lithography VII.

[95] Brainard R., Kruger S., Higgins C., Revuru S., Gibbons S., Freedman D., Yueh W., Younkin T. (2009). Kinetics, Chemical Modeling and Lithography of Novel Acid Amplifiers for Use in EUV Photoresists. J. Photopolym. Sci. Technol..

[96] O’Callaghan G., Popescu C., McClelland A., Kazazis D., Roth J., Theis W., Ekinci Y., Robinson A.P. (2019). Multi-Trigger Resist: Novel Synthesis Improvements for High Resolution EUV Lithography. Proc. SPIE 10960, Advances in Patterning Materials and Processes XXXVI.

[97] Popescu C., Frommhold A., McClelland A., Roth J., Ekinci Y., Robinson A.P.G. Sensitivity enhancement of the high-resolution xMT multi-trigger resist for EUV lithography. Proceedings of the Extreme Ultraviolet (EUV) Lithography VIII.

[98] Robinson A., Popescu C.M., Kazazis D., McClelland A., Dawson G., Roth J., Theis W., Ekinci Y. (2018). High-Resolution EUV Lithography Using a Multi-Trigger Resist. Extreme Ultraviolet (EUV) Lithography IX.

[99] Popescu C.M., Vesters Y., McClelland A., De Simone D., Dawson G., Roth J., Theis W., Vandenberghe G., Robinson A. (2018). Multi Trigger Resist for EUV Lithography. J. Photopolym. Sci. Technol..

[100] Cardineau B., Garczynski P., Earley W., Brainard R. (2013). Chain-Scission Polyethers for EUV Lithography. J. Photopolym. Sci. Technol..

[101] Echigo M., Oguro D. (2009). Development of New Phenylcalix[4]resorcinarene: Its Application to Positive-Tone Molecular Resist for EB and EUV Lithography. Proc. SPIE 7273, Advances in Resist Materials and Processing Technology XXVI.

[102] Owada T., Yomogita A., Kashiwamura T., Kusaba T., Miyamoto S., Takeya T. (2009). Development of Novel Positive-Tone Resists for EUVL. Proc. SPIE 7273, Advances in Resist Materials and Processing Technology XXVI.

[103] Kudo H., Nina N., Sato T., Oizumi H., Itani T., Miura T., Watanabe T., Kinoshita H. (2012). Extreme ultraviolet (EUV)-resist material based on noria (water wheel-like macrocycle) derivatives with pendant alkoxyl and adamantyl ester groups. J. Photopolym. Sci. Technol..

[104] Kulshreshtha P., Maruyama K., Kiani S., Blackwell J., Olynick D., Ashby P. (2014). Harnessing entropic and enthalpic contributions to create a negative tone chemically amplified molecular resist for high-resolution lithography. Nanotechnology.

[105] Oizumi H., Tanaka K., Kawakami K., Itani T. (2010). Development of New Positive-Tone Molecular Resists Based on Fullerene Derivatives for Extreme Ultraviolet Lithography. Jpn. J. Appl. Phys..

[106] Frommhold A., McClelland A., Roth J., Fallica R., Ekinci Y., Robinson A.P. (2016). Optimization and Sensitivity Enhancement of High-Resolution Molecular Resist for EUV Lithography. Proc. SPIE 9776, Extreme Ultraviolet (EUV) Lithography VII.

[107] Frommhold A., Yang D., McClelland A., Roth J., Xue X., Rosamund M.C., Linfield E.H., Robinson A.P. (2015). Novel molecular resist for EUV and electron beam lithography. J. Photopolym. Sci. Technol..

[108] Sharma S.K., Pal S.P., Reddy P.G., Kumar P., Ghosh S., Gonsalves K.E. (2016). Design and development of low activation energy based nonchemically amplified resists (n-CARs) for next generation EUV lithography. Microelectron. Eng..

[109] Oyama T.G., Oshima A., Tagawa S. (2016). Estimation of resist sensitivity for extreme ultraviolet lithography using an electron beam. AIP Adv..

[110] Rathore A., Pollentier I., Singh H., Fallica R., De Simone D., De Gendt S. (2020). Effect of molecular weight on the EUV-printability of main chain scission type polymers. J. Mater. Chem. C.

[111] Trikeriotis M., Krysak M., Chung Y.S., Ouyang C., Cardineau B., Brainard R., Ober C.K., Giannelis E.P., Cho K. (2012). A new inorganic EUV resist with high-etch resistance. Proceedings of the Extreme Ultraviolet (EUV) Lithography III.

[112] Kosma V., Kasahara K., Xu H., Odent J., Ober C.K., Giannelis E.P. (2017). Elucidating the patterning mechanism of zirconium-based hybrid photoresists. J. Micro/Nanolithogr. MEMS MOEMS.

[113] Sortland M., Del Re R., Passarelli J., Hotalen J., Vockenhuber M., Ekinci Y., Neisser M., Freedman D.A., Brainard R.L. (2015). Positive-tone EUV resists: Complexes of platinum and palladium. Proceedings of the Extreme Ultraviolet (EUV) Lithography VI.

[114] Fujimori T., Tsuchihashi T., Minegishi S., Kamizono T., Itani T. (2016). Novel Ultra-High Sensitive ‘Metal Resist’ for EUV Lithography. Proc. SPIE 9776, Extreme Ultraviolet (EUV) Lithography VII.

[115] Li L., Chakrabarty S., Spyrou K., Ober C., Giannelis E. (2015). Studying the Mechanism of Hybrid Nanoparticle Photoresists: Effect of Particle Size on Photopatterning. Chem. Mater..

[116] Grenville A., Anderson J., Clark B., De Schepper P., Edson J., Greer M., Jiang K., Kocsis M., Meyers S., Stowers J. (2015). Integrated Fab Process for Metal Oxide EUV Photoresist. Proc. SPIE 9425, Advances in Patterning Materials and Processes XXXII.

[117] Hinsberg W., Meyers S. (2017). A Numeric Model for the Imaging Mechanism of Metal Oxide EUV Resists. Proc. SPIE 10146, Advances in Patterning Materials and Processes XXXIV.

[118] Zhang Y., Haitjema J., Baljozovic M., Vockenhuber M., Kazazis D., Jung T., Ekinci Y., Brouwer A. (2018). Dual-tone Application of a Tin-Oxo Cage Photoresist Under E-beam and EUV Exposure. J. Photopolym. Sci. Technol..

[119] Sitterly J., Murphy M., Grzeskowiak S., Denbeaux G., Brainard R. (2018). Molecular organometallic resists for EUV (MORE): Reactivity as a function of metal center (Bi, Sb, Te and Sn). Advances in Patterning Materials and Processes XXXV.

[120] Gädda T., Luong N.D., Laukkanen M., Karaste K., Kähkönen O., Kauppi E., Kazazis D., Ekinci Y., Rantala J. (2019). Advanced EUV Negative Tone Resist and Underlayer Approaches Exhibiting Sub-20nm Half-Pitch Resolution. Proc. SPIE 10960, Advances in Patterning Materials and Processes XXXVI.

[121] Mattson E., Cabrera Y., Rupich S., Wang Y., Oyekan K., Mustard T., Halls M., Bechtel H., Martin M., Chabal Y. (2018). Chemical Modification Mechanisms in Hybrid Hafnium Oxo-Methacrylate Nanocluster Photoresists for Extreme Ultraviolet Patterning. Chem. Mater..

[122] Zhou R., Cao M., Tan Y., Neisser M., Xu H. (2025). Polytelluoxane as the ideal formulation for EUV photoresist. Sci. Adv..

[123] Moon S.Y., Kim J.M. (2007). Chemistry of photolithographic imaging materials based on the chemical amplification concept. J. Photochem. Photobiol. C-Photochem. Rev..

[124] Li J.L.H. (2025). Impact of chemical stochastics in extreme ultraviolet photoresists on the pattern quality. AIP Adv..

[125] Koyama M., Imai K., Shirai M., Hirai Y., Yasuda M. (2021). Effects of acid diffusion and resist molecular size on line edge roughness for chemically amplified resists in EUV lithography: Computational study. Jpn. J. Appl. Phys..

[126] Cen J., Deng Z., Liu S. (2024). Emerging trends in the chemistry of polymeric resists for extreme ultraviolet lithography. Polym. Chem..

[127] Xing S., Wang D., Fan Z., Kang W., Wang Q., Wang X. (2026). Regulation and Synthesis of Mixed-Ligand Titanium-Oxide Nanoparticles for 12 nm Nanolithography. Small.

[128] Otsubo Y., Sakai K., Kasahara K., Xu H., Giannelis E., Ober C. (2022). Progress in EUV Photoresists for High-Resolution Patterning. J. Photopolym. Sci. Technol..

[129] dos Santos T., Erdmann A., Robinson A., McClelland A., Popescu C., van Bree J., van de Kerkhof M. (2025). Multi-Trigger Resists: Modeling and Simulation Results. J. Photopolym. Sci. Technol..

[130] Zhang Y., Yu L., Guo J., Zhao Q., Fan Y., Chen Z., Zhang J., Ye S. (2025). Exposure of Hydrogen Silsesquioxane in Electron Beam Lithography. Acs Appl. Mater. Interfaces.

[131] Hwang J., Lee J., Shin H., Ku B., Sim J., Lim J. (2024). Novel EUV Underlayer Design for Metal Oxide Resist Patterning. J. Photopolym. Sci. Technol..

[132] Hansen E., Zhang Y., Kim J., Hinsberg W. (2022). Analytical approach to metal oxide resist modeling: Exposure, bake, and network formation. J. Micro-Nanopattern. Mater. Metrol..

[133] Peng R., Lian P., Chen J., Yu T., Zeng Y., Wang S., Guo X., Hu R., Zhao J., Wu Y. (2025). Lithographic performances of aryl sulfonate ester-modified polystyrenes as nonchemically amplified resists. Ind. Chem. Mater..

[134] Mack C.A., Mueller K., Gardiner A., Sagan J., Dammel R., Willson C. (1998). Modeling solvent diffusion in photoresist. J. Vac. Sci. Technol. B Microelectron. Nanometer Struct. Process. Meas. Phenom..

[135] Pollentier I., Rathore A., Gupta M., De Simone D., Suh H.S. (2022). To bake or not to bake…: The impact of prebake in the EUV resist process. Proceedings of the Advances in Patterning Materials and Processes XXXIX.

[136] Bauer J.J., Drescher G., Silz H., Frankenfeld H., Illig M. (1997). Surface tension and adhesion of photo-and electron-beam resists. Proceedings of the Advances in Resist Technology and Processing XIV.

[137] Kawai A., Nagata H., Takata H.A. (1991). Adhesion between photoresist and inorganic substrate. Jpn. J. Appl. Phys..

[138] Zhang Y.H., Yu H.J., Wang L., Wu X.D., He J.W., Huang W.B., Ouyang C.G., Chen D.N., Keshta B.E. (2024). Advanced lithography materials: From fundamentals to applications. Adv. Colloid Interface Sci..

[139] Jaeger R.C. (2002). Introduction to Microelectronic Fabrication.

[140] Sharma P., Jackson N. (2021). Vibrating Mesh Atomizer for Spin-Spray Deposition. J. Microelectromech. Syst..

[141] Han S., Derksen J., Chun J.H. (2004). Extrusion Spin Coating: An Efficient and Deterministic Photoresist Coating Method in Microlithography. IEEE Trans. Semicond. Manuf..

[142] Yang F., Wu W.H., Wang H.L., Zhai X.Y., Wang X., Li B.X. (2019). Spray Coating Technique for the Thick Photoresist on Structured Wafers. Micronanoelectron. Technol..

[143] Li J., Wang C., Miao T., Guo S., Tong Y., Zhang L. (2025). Airflow-assisted acoustic spray coating for suppressing pinhole defects on photoresist film. Mater. Sci. Semicond. Process..

[144] Zhang L., Wang X., Miao T., Guo S., Tong Y. (2025). Multilayer Uniform Photoresist Coating on Silicon Wafers via Spin-Coupled Inkjet Printing. IEEE Trans. Compon. Packag. Manuf. Technol..

[145] Li J., Wang X., Sun Y., Zhang L. (2024). Photoresist Spray Coating on Silicon Wafers With Acoustic Resonance Atomization. IEEE Trans. Semicond. Manuf..

[146] Mack C. (2007). Fundamental Principles of Optical Lithography: The Science of Microfabrication.

[147] Emslie A.G., Bonner F.T., Peck L.G. (1958). Flow of a Viscous Liquid on a Rotating Disk. J. Appl. Phys..

[148] Liu P., Huang L., Zheng C., Bao Y., Gao D., Zhou G. (2025). Spin coating in semiconductor lithography: Advances in modeling and future prospects. Microelectron. Eng..

[149] Maitra V., Su Y.T., Shi J. (2024). Virtual metrology in semiconductor manufacturing: Current status and future prospects. Expert Syst. Appl..

[150] Wang X., Wang Y., Liu Y., Wei Y., Ye T. (2024). Application of a CFD simulation on spin coating process. Proceedings of the 2024 2nd International Symposium of Electronics Design Automation (ISEDA).

[151] Yan Y., Li J., Liu Q., Zhou P. (2021). Evaporation Effect on Thickness Distribution for Spin-Coated Films on Rectangular and Circular Substrates. Coatings.

[152] Jung J.-Y., Kang Y.T., Koo J. (2010). Development of a new simulation model of spin coating process and its application to optimize the 450 mm wafer coating process. Int. J. Heat Mass Transf..

[153] Liu J. (2023). Fluctuating Hydrodynamics of Nanoscale Thin Films. Ph.D. Thesis.

[154] Craster R.V., Matar O.K. (2009). Dynamics and stability of thin liquid films. Rev. Mod. Phys..

[155] Birnie D.P., Manley M. (1997). Combined flow and evaporation of fluid on a spinning disk. Phys. Fluids.

[156] Zhou P., Liu Q., Zhang Z. (2025). Coupled modeling of solvent evaporation and thin film evolution in spin coating. Appl. Therm. Eng..

[157] Routh A.F. (2013). Drying of thin colloidal films. Rep. Prog. Phys..

[158] Mahmoodi S. (2023). Microstructure and resolution of etched patterns of Photoresist (AZP4620) layers spin-coated under artificially elevated gravity accelerations by two-axis spin coating technology. J. Mater. Sci. Mater. Electron..

[159] Mahmoodi S., Guoqing H., Wang L., Nourikhajavi M. (2017). Two-dimensional spin coating technology and the effect of artificial gravity on film’s air-bubbling. Microsyst. Technol..

[160] Reiter G. (1992). Dewetting of thin polymer films. Phys. Rev. Lett..

[161] Zheng L., Xia Y., Jia X., Gao M., Liu N., Song J., Li X., Zhao X., Gao X., Zhou W. (2025). Cryo-electron tomography reconstructs polymer in liquid film for fab-compatible lithography. Nat. Commun..

[162] Chia C., Martis J., Jeffrey S.S., Howe R.T. (2019). Neural network-based model of photoresist reflow. J. Vac. Sci. Technol. B.

[163] Liu Y.J., Ni D., Shao X., Gong D.L., Li J.J. (2024). A hierarchical model-based method for wafer level virtual metrology under process information deficiency. Qual. Eng..

[164] Chaplick V., Degenkolb E., Elliott D., Harte K., Millman R., Tardif M. (2010). Analysis of photoresist edge bead removal using laser light and gas. Proceedings of the Optical Microlithography XXIII.

[165] Park S., You C. (2023). Deep convolutional generative adversarial networks-based data augmentation method for classifying class-imbalanced defect patterns in wafer bin map. Appl. Sci..

[166] Pang G., Shen C., Cao L., Hengel A.V.D. (2021). Deep learning for anomaly detection: A review. ACM Comput. Surv. CSUR.

[167] Yu N.G., Li H.Z., Xu Q. (2023). A full-flow inspection method based on machine vision to detect wafer surface defects. Math. Biosci. Eng..

[168] Niu S.L., Li B., Wang X.G., Peng Y.R. (2022). Region- and Strength-Controllable GAN for Defect Generation and Segmentation in Industrial Images. IEEE Trans. Ind. Inform..

[169] Hwang J., Noh S. (2024). Digital Twin-Based Optimization of Operational Parameters for Cluster Tools in Semiconductor Manufacturing. IEEE Access.

[170] Qin S.J. (2012). Survey on data-driven industrial process monitoring and diagnosis. Annu. Rev. Control.

[171] Zabrocki S., Jo P.S., Park C., Yim D., Yun S., Lee B.-J. (2023). Adaptive online time-series prediction for virtual metrology in semiconductor manufacturing. Proceedings of the 2023 34th Annual SEMI Advanced Semiconductor Manufacturing Conference (ASMC).

[172] Zhao R., Wang X., Wei Y., He X., Xu H. (2024). Machine Learning Applied to Electron Beam Lithography to Accelerate Process Optimization of a Contact Hole Layer. ACS Appl. Mater. Interfaces.

[173] Shinde P.P., Pai P.P., Adiga S.P., Subramanya Mayya K., Seo Y., Hwang M., Go H., Park C. (2025). Defect detection in photolithographic patterns using deep learning models trained on synthetic data. Heliyon.

[174] Friederich J., Francis D.P., Lazarova-Molnar S., Mohamed N. (2022). A framework for data-driven digital twins of smart manufacturing systems. Comput. Ind..

[175] Emami-Naeini A., Ebert J.L., Kosut R.L., de Roover D., Ghosal S. (2004). Model-based control for semiconductor and advanced materials processing: An overview. Proceedings of the 2004 American Control Conference.

[176] Xie W.P., Wu J.C., Wang Y.P., Chen Y.N. (2025). S2GA-VM: Self-supervised and global-aware virtual metrology for accurate film thickness prediction in semiconductor manufacturing. J. Intell. Manuf..

[177] Fan H., Ren T., Qin X. (2021). Double Coating Process Using the Single Photoresist and the Thickness Prediction. IEEE Trans. Semicond. Manuf..

[178] Li C., Hsieh Y.M. (2025). Sudden Concept Drift Detection and Adaptation in Virtual Metrology for Semiconductor Manufacturing. IEEE Access.

[179] Sivasubramanian C.K., Dodge R., Ramani A., Bayba D., Janakiram M., Butcher E., Gonzales J., Pedrielli G. (2023). DTFab: A digital twin based approach for optimal reticle management in semiconductor photolithography. J. Syst. Sci. Syst. Eng..

[180] Lin C., Tseng T., Tsai T. (2025). A Digital Twin Framework With Bayesian Optimization and Deep Learning for Semiconductor Process Control. IEEE Access.

[181] Moyne J., Del Castillo E., Hurwitz A.M. (2018). Run-to-Run Control in Semiconductor Manufacturing.

[182] Nuhu A., Zeeshan Q., Safaei B., Shahzad M. (2023). Machine learning-based techniques for fault diagnosis in the semiconductor manufacturing process: A comparative study. J. Supercomput..

[183] Lei T., Liu Z., Liu Z., Xue G., Sun C., Zhou J., Miao X. (2025). Optimization and defect control in photoresist etch back processes for advanced semiconductor technologies. J. Semicond..

[184] Gama J., Žliobaitė I., Bifet A., Pechenizkiy M., Bouchachia A. (2014). A survey on concept drift adaptation. ACM Comput. Surv. CSUR.

[185] Doshi-Velez F., Kim B. (2017). Towards a rigorous science of interpretable machine learning. arXiv.

[186] Rudin C. (2019). Stop explaining black box machine learning models for high stakes decisions and use interpretable models instead. Nat. Mach. Intell..

[187] Karpatne A., Atluri G., Faghmous J.H., Steinbach M., Banerjee A., Ganguly A., Shekhar S., Samatova N., Kumar V. (2017). Theory-guided data science: A new paradigm for scientific discovery from data. IEEE Trans. Knowl. Data Eng..

[188] Raissi M., Perdikaris P., Karniadakis G.E. (2019). Physics-informed neural networks: A deep learning framework for solving forward and inverse problems involving nonlinear partial differential equations. J. Comput. Phys..

[189] Melvin L.S., Welling U., Kandel Y., Levinson Z.A., Taoka H., Stock H.-J., Demmerle W. (2022). Applying stochastic simulation to study defect formation in EUV photoresists. Jpn. J. Appl. Phys..

[190] Qiao Y., Li C., Yan F., Liu Z., Wang X., Xie J., Shi G., Wei J., Zhao J., Zhang L. (2025). Synergistic Enhancement Effects of Heterogeneous Isomorphism Clusters in Response to Irradiation: Sub-10 nm Nanolithography and Nanoscale Etching Transfer. Nano Lett..

[191] Hu Z.Q., Wu Y.Q. (2025). Survey of semiconductor wafer defect detection method based on machine vision. J. Image Graph..

[192] Nagy D., Indalecio G., García-Loureiro A., Elmessary M., Kalna K., Seoane N. (2018). FinFET Versus Gate-All-Around Nanowire FET: Performance, Scaling, and Variability. IEEE J. Electron Devices Soc..

[193] Fukuda H., Momonoi Y., Sakai K. (2019). Estimating extremely low probability of stochastic defect in extreme ultraviolet lithography from critical dimension distribution measurement. J. Micro/Nanolithogr. MEMS MOEMS.

[194] Ithakifirooz M., Fathi M., Chien C.F. (2018). Modelling and decision support system for intelligent manufacturing: An empirical study for feedforward- feedback learning-based run-to-run controller for semiconductor dry-etching process. Int. J. Ind. Eng.-Theory Appl. Pract..

[195] Ren J.-C., Liu D., Wan Y. (2021). Model-free adaptive iterative learning control method for the Czochralski silicon monocrystalline batch process. IEEE Trans. Semicond. Manuf..

[196] Medvedev V., Erdmann A., Roßkopf A. (2024). 3D mask simulation and lithographic imaging using physics-informed neural networks. Optical and EUV Nanolithography XXXVII.

[197] Lin Y., Li M., Watanabe Y., Kimura T., Matsunawa T., Nojima S., Pan D.Z. (2018). Data efficient lithography modeling with transfer learning and active data selection. IEEE Trans. Comput.-Aided Des. Integr. Circuits Syst..

[198] He H., Wang Z., Wang J., Wu T., He X., Yu B., Yu J., Geng H. LithoSim: A Large, Holistic Lithography Simulation Benchmark for AI-Driven Semiconductor Manufacturing. Proceedings of the The Thirty-Ninth Annual Conference on Neural Information Processing Systems Datasets and Benchmarks Track.

[199] Lookman T., Balachandran P.V., Xue D., Yuan R. (2019). Active learning in materials science with emphasis on adaptive sampling using uncertainties for targeted design. npj Comput. Mater..

[200] Kusiak A. (2018). Smart manufacturing. Int. J. Prod. Res..

[201] Sandru E.D., David E., Kovacs I., Buzo A., Burileanu C., Pelz G. (2022). Modeling the Dependency of Analog Circuit Performance Parameters on Manufacturing Process Variations With Applications in Sensitivity Analysis and Yield Prediction. IEEE Trans. Comput.-Aided Des. Integr. Circuits Syst..

[202] Tao F., Qi Q., Liu A., Kusiak A. (2018). Data-driven smart manufacturing. J. Manuf. Syst..

[203] Sculley D., Holt G., Golovin D., Davydov E., Phillips T., Ebner D., Chaudhary V., Young M., Crespo J.-F., Dennison D. (2015). Hidden technical debt in machine learning systems. Advances in Neural Information Processing Systems 28.

[204] Kairouz P., McMahan H.B. (2021). Advances and open problems in federated learning. Found. Trends Mach. Learn..

[205] Denbeaux G., Azhari N., Ai R.W., Kahl B., Painter M., Adamson Z., Aldrin A., Dilina E., Choudhary A., Brainard R.L. (2023). Understanding the onset of EUV resist chemical stochastics. Jpn. J. Appl. Phys..

[206] Kozawa T., Santillan J.J., Itani T. (2013). Analysis of stochastic effect in line-and-space resist patterns fabricated by extreme ultraviolet lithography. Appl. Phys. Express.

[207] Stillwagon L., Larson R. (1988). Fundamentals of topographic substrate leveling. J. Appl. Phys..

[208] May G.S., Spanos C.J. (2006). Fundamentals of Semiconductor Manufacturing and Process Control.

[209] de la Rosa F.L., Gómez-Sirvent J.L., Morales R., Sánchez-Reolid R., Fernández-Caballero A. (2022). A deep residual neural network for semiconductor defect classification in imbalanced scanning electron microscope datasets. Appl. Soft Comput..

[210] Lee J., Bagheri B., Kao H.-A. (2015). A cyber-physical systems architecture for industry 4.0-based manufacturing systems. Manuf. Lett..

[211] Weber A. (2020). Smart manufacturing in the semiconductor industry: An evolving nexus of business drivers, technologies, and standards. Smart Manufacturing.

[212] Kritzinger W., Karner M., Traar G., Henjes J., Sihn W. (2018). Digital Twin in manufacturing: A categorical literature review and classification. IFAC-PapersOnLine.

[213] Das R.R., Rajalekshmi T.R., James A. (2024). FinFET to GAA MBCFET: A Review and Insights. IEEE Access.

[214] Suthar K., Shah D., Wang J., He Q.P. (2019). Next-generation virtual metrology for semiconductor manufacturing: A feature-based framework. Comput. Chem. Eng..

[215] Santillan J.J., Shichiri M., Itani T. (2015). The effect of resist dissolution process on pattern formation variability: An in situ analysis using high-speed atomic force microscopy. Proceedings of the Advances in Patterning Materials and Processes XXXII.

[216] Neureuther A.R., Long L., Naulleau P. (2021). Modeling stochastic effects of exposure/diffusion and dissolution on missing contacts. Proceedings of the Extreme Ultraviolet (EUV) Lithography XII.

